# Analectic

**Published:** 1834

**Authors:** 


### MISCELLANEOUS INTELLIGENCE.

### ANALECTIC, ANALYTICAL, AND ORIGINAL.

### I. Analectic.

*Ad Sylvas Nuncius.*

1. *Confirmation of Sir Chas. Bell's Opinions on the Functions of the Anterior and Posterior Fasciculi of the Spinal Nerves.*—Our attention has been recently drawn to a very valuable paper of Professor Muller, of Bonn, in a late number of the Annales des Sciences Naturalles. The experiments which he adduces are most satisfactory, and will be no doubt considered conclusive, even in Germany, where the doctrine of the separate functions of the abdominal and dorsal roots of the spinal nerves has not been altogether assented to. Meckel, Rudolphi, Weber, and others have admitted it, only as conjectural; our author himself performed some experiments in 1824, with the view of ascertaining its correctness, but the results were far from being uniform and decisive. Bellingeri, in Italy, was also engaged about that time in similar researches, and the conclusions which he drew were, that the anterior fasciculi presided over the sensibility, and the flexion movements of the trunk and extremities, while the posterior presided over the movements of extention. Even Magendie, to whom the second seat of honor is due, as a physiologist of the nervous system, and along with him Desmoulins, in their Anatomie des Syst. Nerv. have not assigned totally exclusive functions to the two sets of nerves in question. Their own words are—"Sl l'on galvanise l'une aprés l'autre, une racine dorsale, et une racine abdominale, qui ne communique plus avec la moelle, on obtient à la verité des contractions par chaque racine. Mais les contractions par les racines anterieures sont en general plus fortes, et plus completes, que par les racines posterieures. Les racines posterieures pincées, tiraillées, piquées, causent de la douleur, mais une douleur bien moindre, que celle qui result de l'irritation de la partie correspondante de la moelle. Alors aussi les muscles correspondans aux nerfs, dont on irrite une racine, se contractent; mais ses contractions sont encore moindres que dans le cas de l'irritation méme de la moelle. La section d'un faisceau de racines dorsales cause une secousse de tout le membre correspondant. Les resultats sont inverses en operant sur les racines abdominales; leurs figures, leurs pincemens, produisent des contractions plus fortes et convulsives tandis que les signes de douleur sont presque nuls.—L'isolement des deux propriétés dans chacun des ordres de racines *n'est pas donc absolu.*" Muller commenced a new set of experiments on rabbits, in order to determine this most interesting question; but he found that the previous operation of opening the vertebral canal was so difficult, and attended with such excessive pain to the animals, as frequently to induce involuntary twitches of all the muscles even when the nerves were not directly irritated, so that he was precluded from deducing any satisfactory conclusions. Indeed there must always be this strong objection to all trials made on the higher animals; but the happy thought of Muller, to examine the spinal system of the frog, has fully compensated for the uncertainty of these. The vertebral canal of the frog may be opened with very little trouble, and with comparatively trifling pain; the animal is so tenacious of life, that it remains quite lively after the operation, and the peculiar arrangement of the anterior and posterior fasciculi of nerves, further facilitates our investigations, for these continue to be distinct from each other, and easily separable for a considerable distance from their points of origin; the posterior root may therefore be raised on a needle and submitted to experiment, while the anterior one is free from all injury. We shall first mention the effects of simple mechanical, and then those of galvanic irritation on the two sets of nerves.

1. When the posterior root is divided the animal appears to experience "quelque douleur;" if the distal, or unattached portion be now seized and irritated, there is not the slightest trace of movement in any of the muscles of the trunk, or of the extremities. When the anterior, or abdominal root is simply touched, convulsive movements of the extremities immediately follow. The same phenomenon, only more violent, are observed when this root is cut and irritated.

2. The galvanic experiments were performed at first with a single pair of zinc and copper plates. Upon applying the two plates to cut ends of the anterior roots, the muscles became convulsed; but no such effect was ever produced when they were applied to the posterior roots. This latter position contradicts therefore the assertion of Magendie and Desmoulins; but we must remember that their experiments were performed on mammiferous animals; and in these the two sets of roots are too short to enable us to separate them satisfactorily from each other, and thus to avoid the irritation of one set, while we are experimenting upon the other. Even in the case of the frog, it is necessary, for the sake of accuracy, to isolate the one from the other by means of the small glass plates; because the galvanic irritation of the motor nerves is found to take place at the distance of half a line. But in order to insure perfect accuracy, it is better to employ a small voltaic pile; for then we may either apply both poles to the cut end of the nerve, or we may apply one there, and the other to some of the muscles. The following are the results of Muller's experiments in this way.

1. When the two poles are applied to the posterior roots, no convulsive movements follow. 2. When one pole is applied to the posterior nerve, and the other to some muscle at a distance, slight movements of the muscles which are situated in the tract of the galvanic current are observed. 3. When the anterior root is made the subject of these experiments, convulsive movements immediately occur, whether both poles are applied to the nerve, or only one, the other being applied to a muscle; and these movements take place not only in the muscles which are situated in the tract of the current, but throughout the whole extremity. 4 The same result, viz. the occurrence of convulsions, is obtained when one pole is applied to the posterior, and the other to the anterior root. We may therefore safely draw the conclusions, that the posterior roots of the spinal nerves never directly and of themselves provoke muscular contraction; that when they seem to do so (as in the 2d result) it is only from their acting as conductors, just in the same way as any other moist animal substance, of the galvanic current; and lastly that the anterior nerves not only are conductors of the galvanic current, but also are excited thereby to induce muscular movements in the direction of their branches. Now one of these anterior nerves may be deprived of its "vis motoria," and yet retain its conducting power:—to exhibit this, we need only seize and compress it firmly at a little distance from the cut end; and we shall find that no irritation, either mechanical or galvanic, applied between the point of compression and this end will induce any contractions, but if one of the galvanic poles be applied to the end and another to a distant muscle to which the nerve is distributed, then contractions will immediately follow, just as if there was no intermediate pressure; shewing thereby most distinctly that the nerve retains its conducting power.

It has been supposed that galvanism acts as a special and peculiar irritant to the nerves, and in a manner altogether different from mere mechanical injury; but this is not true, for any foreign body, even not metallic, such for example as a quill, when applied to a motor nerve, will provoke muscular contractions. Muller, from multiplied observation, has been led to conclude,—1, that galvanism acts upon the nerves like any other extraneous agent—2, that it is not the proximate cause of muscular contraction; but only that it irritates the nerves, and provokes their "vis motoria," which is altogether different from a galvanic power—3, that it has been proved that nerves are better conductors of galvanism than other moist animal substances—4, that galvanism excites movements, only when a muscle or a motor nerve are situated in the tract of its current—5, that there are some nerves which have no moving power, and can never of themselves induce any movements; that these are only passive conductors of galvanism—6, that there are other nerves which induce muscular movements, not only on the application of galvanism, but also of any mechanical irritant—7, that the dorsal or posterior roots of the spinal nerves have no "vis motoria," but that the anterior have, and that, from these last, all the motor fibres of the conjoined spinal nerves are derived. He once more alludes to the fallacy of believing that the posterior are ever motor nerves, merely because, when one pole is applied to them and the other to a muscle, certain movements take place.

The next object of his investigation, was to ascertain what effects are produced by irritation of the proximal, or attached ends of the divided anterior and posterior roots. He found that, when a mechanical agent, or when both poles of a galvanic apparatus, were applied to any of these, no muscular movements were ever induced; but that, when one pole was applied to the portions of the roots adhering to the extreme parts (cauda equina?) of the spinal marrow, and the other to some anterior part of the body, as, for example, the head, the muscles of the trunk and extremities were thrown into convulsions. In one experiment, he divided all the anterior and posterior roots as high as the cervical portion of the marrow, and then gently lifted out the spinal cord from its canal, and laid it upon a small glass plate; upon applying both poles to its sacral extremity, there were movements in all the parts which had been left connected with the marrow, viz. the neck and anterior extremities. If this position be confirmed, it would show that the spinal cord is not to be considered as only the "ensemble" o, the nerves which issue from it; for we have seen that the portions of the roots which may be left adhering to the extreme parts of the marr row, do not, upon any irritation, induce muscular movements, but tha- the marrow itself, if irritated, does. t

A few cursory remarks on some of the cerebral nerves are appended to the preceding valuable memoir. Muller agrees with Mayo and others, that the portio dura is not solely and exclusively a motor nerve; when irritated, the animal seems to experience pain. The infra-orbital nerve is one of mere sensation—it has no "vis motoria." With regard to the nerves of the tongue, Muller is led, by his experiments, to state that the lingual, or ninth cerebral nerve, when irritated, or galvanised, provokes violent convulsions of the member; that the gustatory, or third division of trigeminus, excites none of these phenomena, either by mechanical or galvanic agency, except, indeed, when one pole is applied to the nerve and another to the tongue; but, as we have explained before, this sign is quite fallacious, the nerve serving only as a conductor. The glossopharyngeal nerve, on the application of both poles, excites convulsions in the pharynx. These experiments accord with those of Desmoulins and Magendie.

When the lingual nerve was cut, the animal (a cat) seemed to suffer pain; and hence Muller believes that this nerve, although chiefly a motor, is also, in some degree, a sensitive nerve, in the same manner as he supposes the portio dura and par vagum to be. But his experiments here appear to us far from satisfactory.—*Medico-Chirurgical Review, January,* 1834.—*From Annales des Sciences Naturelles.—From Balt. Med. & Surg. Journal & Review.*

2. *The Anatomy and Physiology of the Liver, by* Frances Kiernan, Esq. M. R. C. S.—After giving a short account of the descriptions of Malpighi and other writers respecting the minute structure of the liver, the author proceeds to state the results of his own investigations on this subject. The hepatic veins, together with the lobules which surround them, resemble in their arrangement the branches and leaves of a tree; the substance of the lobules being disposed around the minute branches of the veins like the parenchyma of a leaf around its fibres. The hepatic veins may be divided into two classes; namely, those contained in the lobules, and those contained in canals formed by the lobules. The first class is composed of interlobular branches, one of which occupies the centre of each lobule, and receives the blood from a plexus formed in the lobule by the portal vein; and the second class of the hepatic veins is composed of all those vessels contained in canals formed by the lobules, and including numerous small branches, as well as the large trunks terminating in the inferior cava. The external surface of every lobule is covered by an expansion of Glisson's capsule, by which it is connected to, as well as separated from, the contiguous lobules, and in which branches of the hepatic duct, portal veins, and hepatic artery, ramify. The ultimate branches of the hepatic artery terminate in the branches of the portal vein, where the blood they respectively contain is mixed together, and from which mixed blood the bile is secreted by the lobules, and conveyed away by the hepatic ducts which accompany the portal veins in their principal ramifications. The remaining blood is returned to the heart by the hepatic veins, the beginnings of which occupy the centre of each lobule, and when collected into trunks pour their contents into the inferior cava. Hence the blood which has circulated through the liver, and has thereby lost its arterial character, is, in common with that which is returning from the other abdominal viscera, poured into the vena portæ, and contributes its share in furnishing materials for the biliary secretion. The paper is accompanied by numerous drawings of preparations made by the author, of the minute structure of the liver, in which the different sets of vessels and ducts were injected in various ways.—*London Medical Gazette, Jan.* 1834.—*Balt. Med. and Surg. Journal and Review.*

3. *On the efficacy of the Secale Cornutum in Leucorrhœa.* By G. Negri, M. D.—On the employment of the secale cornutum, and on its efficacy on leucorhœa we shall limit ourselves to some general remarks, which are the result of our experience on this subject, without entering into any detail of the singular cases which occurred under our observation.

Although the secale cornutum will be found one of the most valuable remedies in the simple form of leucorrhœa, even of a very long standing, and which have resisted many other means, still its efficacy in this kind of diseases is not so rapid as in hæmorrhages. This would have been almost expected as a matter of course, from the more chronic character of the former complaint. Therefore we found it more, convenient, and we may even say more safe, to give it in small doses as five or six grains two or three times a day, rather than in larger and more frequently repeated ones. Thus the remedy may be continued for a long period without any inconvenience, and with regular advantage. In leucorrhœa as in menorhagia, we must remember, that the ergot of rye has also a peculiar power over the fibrous texture of the womb, and that pains and spasmodic contractions of this organ may be induced, and then symptoms of metritis, and even an increased discharge, may eventually take place. Then it is of the utmost importance, in leucorrhœa also, to allay any state of inflammation, or of local irritation, by those therapeutical means, which may be required by the particular symptoms of each case, before we have recourse to the secale cornutum.

We find in practising, that some patients could not take at first any dose of this remedy without severe pains being induced in the uterine system, when, after having used other remedies for a certain time, they could take the secale again without the least inconvenience, but, on the contrary, with a decided and progressive advantage on their general state of health.

In one of these patients the os uteri was partially open and indurated, and very tender on the left side of its margin: when the finger pressed over this point, acute pains were excited, darting from that part to the right illiac region. We used in this case the extract of conium with the sulphate of iron, with great benefit, and after this morbid sensibility was subdued, we gave again the secale cornutum for the remaining leucorrhœa with decided benefit, and without any more inconvenience, although continued for a long time. We have lately seen this patient, and her general state of health has wonderfully improved; she feels a great deal stronger, and the white discharge is almost entirely gone; we confidently expect to see her in a short time cured by the ergot of rye, which now she only takes twice a day.

Out of ten cases of leucorrhœa, of which we kept regular notes, the ergot of rye has failed in three. But, in all probability, that happened more from want of experience in the judicious employment of the remedy rather than from its inefficacy.

Of these three unsuccessful cases, two were cured afterwards by other remedies; but one had never been permanently well, either by the ergot of rye or by any other means employed for a long time, both by ourselves and several other practitioners. In this singular case, the secale cornutum appeared to have induced once menorrhagia, after which the patient was better from the white discharge for a little while. Amongst the other things we tried repeatedly the injection of nitrate of silver, as recommended by Dr. Jewel, but with out any good effect, and as it appeared to this gentleman very extraordinary, we recommended her to the doctor himself, but we do not know the result.

The secale cornutum has been successfully employed in leucorrhœa by our colleagues at the St. John's Dispensary, and our friend Dr. Ryan has even used it in private practice with the greatest advantage.

*On the effects of the Secale Cornutum in Gonorrhœa.*—About the modus operandi of the secale cornutum in the above classes of diseases,

Dr. Spajrani expressed his opinion in the following way, leaving however this subject for subsequent inquiries. "I am," says he, "rather inclined to believe, that this remedy does not act either as an astringent, or as a stimulant, but more as a sedative on the capillary vessels, and for this reason it may be conveniently used in *certain* instances of active hæmorrhage and of vascular congestion, where exists a state *approaching very much to inflammation;* but yet *it is not to be used* in instances where some *acute inflammation is present,* for which stronger means must be employed."

With the view of ascertaining these therapeutical principles, and from the advantage already obtained by the ergot of rye in leucorrhœa, we thought we should not incur any great risk by trying it also in gonorrhœa, at first in females, and then, if not injurious, in males. It is true that the preternatural secretion of the mucous membrane of the genital organs in gonorrhœa, is induced by a specific virus, but still we readily believe that its essential pathological character is inflammatory. Therefore no better opportunity could be obtained for ascertaining the supposed modus operandi of the secale cornutum, than to use it in a disease of acknowledged character, and in which we could actually see the effects which might be induced by it.

The following cases will give an idea of the result of our inquiries on this subject.

Case I.—Mary C., married, admitted to St. John's Dispensary on the 9th of May, 1833. She has been ill with gonorrhœa for about three weeks; she caught the disease from her husband, and had been under our care some months ago for a similar complaint, induced by the same cause. She complains of shooting pains through her womb and loins, with ardor urinæ. She has been regular three weeks ago, and has never been subject to leucorrhœa. An opening medicine was ordered, and she was directed to take afterwards six grains of the secale three times a day.

May 13th. She is a great deal better; has now no discharge; had no giddiness, but only pains in the lower part of the abdomen, and a kind of cramp of the womb; feels still pain in making water. The same powders to be taken only night and morning.

20th. No discharge; complains still of shooting pains in the womb; secale suspended, and only supertartrate of potash to be taken as an imperial drink. On the 29th she was taken unwell, but the catamenial discharge was very scanty and pale, after which, on the 6th of June, had a slight return of the discharge, which was gradually arrested by the secale in moderate doses. She was discharged cured on the 25th of July.

Case II.—Mary Anne C., æt.; 26, single, admitted on the 9th of May, 1833.

Has had gonorrhœa for nearly two months; has not been regular for several months, and has been subject to leucorrhœa; bowels regular. Six grains of the ergot of rye were ordered to be taken every four hours.

13th. The discharge ceased after having taken four or five powders, and has not returned since: proved no inconvenience by taking her powders. They were ordered to be taken only night and morning.

30th. She menstruated on the previous day, and was left without medicine.

June 6th. Has no discharge at all, and says she is quite well.—Discharged cured.

Case III.—Harriet R., æt. 27, married, admitted on the 20th of May, 1833.

Has had gonorrhœa four years ago, from which she was perfectly cured. She was taken ill again with the same complaint, caught from her husband about ten weeks ago, for which she has been treated, as an out patient at St. Bartholomew's Hospital, under Mr. Lawrence. Balsamic medicines and mercurial pills were given to her, from which she was much relieved. Now the discharge is thin and white, when before it was yellow and thick. Complains still of some starting pains through the womb, but has less pain in making water; complains of pain in her right leg, where there is inflammation of the periosteum on the shin bone, probably of a syphilitic character; her bowels being costive, a cathartic powder was ordered, and five grains of the secale cornutum, to be taken every four hours, beginning the following morning.

23d. The discharge is less; she feels sick, after having taken her powder, and complains of being very weak.—Pergat.

30th. The discharge is less than the preceding day of attendance; she has now no pain in making water, but continues to feel sick after taking the powder; has had no giddiness. Continue the powder three times a day.

June 6th. The discharge has ceased. The secale was suspended, and the mercurial treatment was adopted for what we thought a syphilitic complaint.

Case IV.—John F., æt. 40, a baker, admitted on the 21st of June, 1833.

Has had gonorrhœa about six times; it usually resisted every remedy, and once he had it for nine months; now he has had gonorrhœa for about three weeks; has great pain and scalding in making water, and generally some drops of blood follow; has a great deal of discharge, and the orifice of the urethra is reddened and swollen; in the night he has painful erections. Five grains of the secale cornutum to be taken every four hours.

22d. We saw again the patient. He has taken five powders; the discharge is not abated, but he thinks he has less pain in making water.—Pergat.

28th. He has taken about sixteen powders; he has no pain in making water; he has had still painful erection at night, but the orifice of the urethra is a great deal less red, and the discharge is considerably abated; he has now no inconvenience, except a very slight feeling of warmth in making water. He continued the medicine in ten grain doses in every four hours, until the 11th of July, when he was nearly well, and requested to be discharged.

The patient was under Dr. Ryan's care, and was repeatedly seen by ourselves and colleagues.

Case V.—William M., æt. 24, admitted on the 22d of August, 1833. Has had gonorrhœa about twelve months ago; has now been ill, for the second time, with the same complaint for a fortnight; discharge copious, yellow and thick. Five grains of the secale cornutum to be taken every three hours.

26th. Is just the same but does not feel worse.—Pergat.

September 2d. He is a great deal better.—Pergat.

12th. Discharge scarcely perceptible. Continue the powders, but only one, three times a day.

16th. Discharge almost gone.—Pergat.

He went on taking his powders till the 10th of October, when he asked for another dose of them, to be taken night and morning, having still some little discharge only in the morning. This patient who was very attentive, and appeared much satisfied with his powders, having not returned, we have good reason to believe he is now doing well.

Case VI.—Wm. S., æt. 28, admitted on the 4th of September, 1833. Was taken ill with gonorrhœa a week ago; feels great pain in making water; discharge copious, yellow and thick.

℞. Pulv. secalis cornuti gr. v. 3a. q. h. s.

16th. Discharge thinner; pain in making water gone.—Pergat.

23d. Discharge increased; secale suspended, and prescribed the mistura balsamica.

October 7th. Discharged cured.

Case VII.—Only a few days ago we had in private practice a patient affected with gonorrhœa for the first time. The symptoms were not severe; the discharge moderate. Being an individual of a delicate constitution, and of very regular habit, we expected to do some good in this case with secale cornutum. Three grains of Battley's extract were ordered to be taken every three hours. The following day the discharges appeared a great deal less, and the remedy was continued. Two days afterwards the discharge increased, as well as the ardor urinæ, and he had painful nocturnal erections. The remedy was brought to five grains every three hours, but was soon afterwards suspended, and other means adopted. In this case the secale cornutum certainly increased the severity of the symptoms; and the discharge which was moderate at first, and thin, became afterwards, copious, thick, and sometimes tinged with blood. The pulse was also feverish and sharp, the skin warmer than naturally. This patient is usually of a costive habit, but has a great aversion to take purgatives, had we used them previously, or simultaneously with the secale, we could perhaps have obtained a better result. This was necessarily done after we had resorted to the other usual means generally employed for that complaint.

### Short Notes of Cases by Dr. Ryan.

Case VIII.—"M. M., æt. 22, married, has contracted gonorrhœa from her husband; became a patient under my care at St. John's Dispensary, Sept. 12, 1833. Is two months ill. She was ordered ʒ iss of secale, in twelve powders, one to be taken three times a day.

"16th. Discharge nearly gone. To continue.

"23d. Discharge has entirely ceased."

Case IX.—"Charles C., æt. 22, has suffered from gleet for six months. Commenced the secale Sept. 17th, 1833, and on the 23d was nearly well. He has taken a variety of medicines, but nothing stopped the discharge so rapidly as the powders."

Case X.—"G. S. æt 34, has been six months affected with gleet.—Commenced the secale Oct. 30th, 1833. Took fourteen powders, without any benefit. This was a morning patient, and had the medicine of a druggist, which, perhaps, was bad."

Case XI.—"A. B., æt. 34, has suffered from gleet for eight months. He was cured by twelve doses of the secale cornutum."

Case XII.—"J. A. L., æt. 19, applied to me Nov. 5th, 1833. Has gonorrhœa for the first time; symptoms severe. Ordered the secale.

"8th. Discharge more copious, ardor urinæ severe.—Secale omitted.

"Ordered carbonate of soda in barley water or linseed tea.

"In this case I did not expect much benefit from the secale, but was resolved to try it. Every medical practitioner is aware, that a first gonorrhœa is much more severe and indomitable than when the patient has had the disease frequently, or when the acute symptoms have ceased. But as I have known cubebs repeatedly arrest gonorrhœa in the acute stage, I saw no objection to employ the secale cornutum."

From the above facts it appears to us quite evident, that the secale cornutum has a peculiar action on the mucous membranes; but if exhibited when there is a state of acute inflammation, their morbid secretion may be considerably increased. On the contrary, when a more chronic form of inflammation exists, the secale cornutum may have a beneficial influence in arresting their preternatural discharge.

These deductions being in perfect accordance with what has been already remarked on the efficacy of ergot of rye in hemorrhages and leucorrhœa, we think Dr. Spajrani's assertions on this point pretty correct. If any sedative or anti stimulant property on the capillary vessels of the mucous membrane may be ascribed to the secale cornutum, as Dr. Spajrani is inclined to believe, we do not know. It is true, that in case IV. of gonorrhœa, where did exist redness and swelling round the orifice of the urethra, this appearance subsided under the influence of the secale; and that, in some instances of hemorrhage, the patients were complaining of a general prostration and faintness; but others, on the contrary, felt stronger, and their pulse appeared to us more excited; when, in cases VII. and XII. of gonorrhœa, the inflammatory symptoms were considerably increased. Are some of the former symptoms to be ascribed to the narcotic influence on the nervous system, rather than to any real sedative property of this remedy? We are inclined to believe so. We were much pleased in finding out that MM. Trousseau and Maisonneuve are of the same opinion on this point. (See *Lancet, March 30th,* 1833.

Now, to give a more satisfactory idea of our results on the employment of the secale cornutum in hemorrhage and leucorrhœa, we put down all the different instances of both in the following

*Tabular Form indicating the result of all the Cases of Hemorrhages and Leucorrhœa which came under our observation, from the* 16*th of April,* 1832, *to the* 4*th of November*, 1833, *and were treated with the Secale Cornutum:*

| Different Forms of the Disease. | Total number of Cases. | Successful Cases. | Unsuccessful Cases. | Remarks. |
|---|---|---|---|---|
| Menorrhagia. | 12 | 8 | 4 | |
| Hemorrhage from the rectum. | 2 | 2 | — | *We include in the whole number the cases treated by Doctor Macmichael, at the Middlesex Hospital.<br>† We put down as an unsuccesful case the first attack of the disease of Elizabeth Pilcher [case VI.] although in the second, the secale turned out very beneficial when given in proper doses. |
| Hæmatemesis. | 4* | 3 | 1† | |
| Epistaxis. | 1 | 1 | — | |
| Hæmoptoe. | 1 | 1 | — | |
| Leucorrhœa. | 10 | 7 | 3 | |
| Total. | 30 | 22 | 8 | |

*Note.*—We did not put down in this table the cases of gonorrhœa because they were related merely to show the effects, and not the efficacy, of the secale cornutum on that disease. All we can say, from the limited number of observations on this subject, is, that perhaps the ergot of rye may be found of some service in the more chronic form of that disease.

Of the unsuccessful cases of menorrhagia, the first was a woman who had a copious loss of blood from the vagina, with great tenderness at the lower part of the abdomen, and pains round the groins and loins. Her pulse was such as would have induced us to bleed her, had we not wished to try the secale cornutum in this case, which was the second that had come under our observation, since we began to employ this remedy. Five grains of the secale were ordered to be taken three times a day. The powders were taken for two days, and the pains and loss of blood were considerably increased. They were suspended; she was bled from the arm, and astringent medicines were ordered, which cured her very soon. This unfavorable result led us to adopt another method of practice in the following case (the second related to the society,) which succeeded very well, viz: to bleed first, then to give the secale.

The second was a stout woman who at her regular period was taken ill, but the bloody discharge was very profuse, and went on more or less for nine weeks. Had great pain at the lower part of the abdomen, and round her loins. Her pulse appeared weak. Five grains of the secale were ordered to be taken every second hour. Three days afterwards she was not better, and felt an increase of the pains after taking her powders. Her pulse was stronger. She stated having had a miscarriage about three months ago. She was bled, and directed to take the secale only three times a day, from which she felt worse, and was then suspended, and other means adopted.

The third case was that of Sarah Jones, æt. 33, married, admitted September 2nd, 1833.

Has had five children and miscarried twice, the second time five weeks ago, when she lost a great deal of blood. Three weeks afterwards, finding herself better, started for some place in the country, and came home to London, a distance of eighty miles. She was taken ill again on her journey, and lost a great deal of blood. She continued so more or less till August 31st. On the first of September the hemorrhage became very violent, and she came to our dispensary the following day, and was under our care. She does not complain of any great pains but in her left iliac region and loins; her complexion is very pale, and there is a great action of the heart and arteries, but her pulse is certainly weak and empty; her head feels heavy; her bowels are costive.

℞. Pulv. secalis cornuti, ʒ iij;
Divide in pulv. xxiv;
Pulv. i. 3a, q. h. sumend.; viz. about vij. grains every three hours. A mixture with a drachm of carbonate of magnesia and two scruples of rhubard in six ounces of water, was also given, a wineglassful of which to be taken every night, or night and morning, if her bowels were not open.

September 5th. She was sick, and vomited twice after taking the first powders but felt only a sense of sickness afterwards at each time she took her powders. Had some giddiness, but the pains in her loins and side were relieved. The bloody discharge is reduced very much, and she states that it was so soon after having taken a few powders. Bowels regularly open. Soon after having taken a powder she feels "*a general sense of weakness all over, from the head to the tips of the fingers and toes, as she could not stand; then she feels sick.*" Action of the heart and arteries less violent. Pulse more natural and soft. The mixture to be continued, and the powders to be taken only every five hours.

September 12th. She is better; discharge a great deal diminished, and less colored. The powders continued to make her sick and weak. Continue with the powders.

16th. Feels very sick with her powders. Discharge a great deal increased; but she thinks *her time to be unwell is very near.* The secale was discontinued, and she was gradually doing well under the use of the following pills:

℞. Ferri sulphatis, gr. i;
Extract. rhei, gr. iij;
M. f. pil. ter die sumend.

These pills were continued till the 7th of October, when she was discharged cured. Under these remedies the palpitation of the heart, and the excessive arterial action were reduced to their natural standard, and the patient got very soon better from that sense of general prostration, of which she so much complained before.

Although we put down this with the *unsuccessful cases,* still we thought that properly speaking, it should not have been considered entirely so, for the hæmorrhage increased in consequence of her having taken the secale when near menstruation. This was the reason which induced us to say in another place, that "only in two or *three* cases of menorrhagia, the loss of blood, &c. were remarkably increased by the action of the remedy."

The fourth unsuccessful case of menorrhagia is that of Mary Ann May, æt. 22, married, admitted the 4th of November, 1833. She had miscarried a short time ago, and was laboring under profuse menorrhagia for several days; was complaining of pains in the lower part of the abdomen and loins. She is a thin and delicate looking woman; her pulse appeared to us rather weak. Five grains of the secale cornutum were ordered to be taken every two or three hours.

Nov. 7th. She has been a great deal worse. The hæmorrhage increased very much, with spasmodic pains in the hypogastric region, and had giddiness, and pains along her thighs and legs. She took only six powders, and as soon as she left them off, the pains decreased.—Her pulse was quick, and sharp, but empty. Her bowels are rather costive. The following mixture was ordered:

℞. Magnesiæ sulphatis ℥ j;

Antimonii tartarisati, gr. ij;

Aque fortis, ℥ viij;

M. Cyath. parvul. i. bis terve die sumend.

21st. After the first glass of her medicine, she vomited several times, after which the hæmorrhage suddenly ceased, and she felt a great deal better; this was the reason she did not attend regularly. Now the hæmorrhage returns a little if she has to exert herself too much. The secale cornutum was ordered again to be taken only two or three times a day.

25th. The hæmorrhage *entirely ceased* last Thursday evening (Nov. 21st) after having taking one of her powders which she continued taking till to-day. She felt some pain in the lower part of the abdomen, but a great deal less than at the time she took them.*

In using the secale cornutum we preferred to give it in powder, as Doctor Spajrani did, being also the most economical and convenient way in dispensary practice.

---

* This case, which came under our observation some time after we had written the first part of this paper, was not there mentioned.—We have put it then amongst the unsuccessful cases, although it was only from our injudicious employment, and not from inefficacy of the remedy, that the hæmorrhage had not been arrested at first.

*Tabular Epitome of all the Cases of Hæmorrhage and Leucorrhœa which came to our knowledge, since Dr. Spajrani's publication, cured by the Secale Cornutum by different Practitioners in Italy, France, and England.*

| Where, and by whom treated. | Total number of cases. | Different kind of Hæmorrhages. From the Womb. | From the Nose. | From the Chest. | From the Bladder. | From the Stomach. | From the Rectum. | From the Gums. | Leucorrhœa. | Where published or recorded |
|---|---|---|---|---|---|---|---|---|---|---|
| *In Italy.* | | | | | | | | | | |
| Dr. Spajrani | 17 | 8 | 2 | 5 | | — | — | — | not† sta'd | Omedei's Annali di Medicina e Chirurgia for Mar. 1830. Lancet, Feb. 5, 1831. |
| Dr. Pignacca | 4 | 2 | — | 2 | — | — | — | — | — | Do. Number for May and Jnne, 1830: Lancet, do. |
| Dr. Gabini | 7 | 3 | 2 | 1 | — | 1 | — | — | — | Do. Number for Feb. and March, 1831. |
| Dr. Bazzoni | 8 | — | — | — | — | — | — | — | 8 | Do. do. |
| *In France.* | | | | | | | | | | |
| M. Trousseaux et Maisoneuve | 13 | 13 | — | — | — | — | — | — | — | Bulletin General de Therapeutique. Lancet, March 30, 1833. |
| *England.* | | | | | | | | | | |
| Dr. M. Hall | 1 | — | — | — | — | — | — | — | not sta'd | London Medical and Physical Journal for March, 1829. |
| Dr. Lanyon | 1 | — | — | 1 | — | — | — | — | — | Lancet, for March 13, 1833. |
| Mr. Bright | 1 | 1 | — | — | — | — | — | — | — | Do. for April 13, 1833. |
| Mr H A O'Slea | not‡ sta'd | | | | | | | | | Do. do. |
| Dr Macmichael | 1 | — | — | — | — | 1 | — | — | — | Dr. Negri's Paper. (See Case vii. |
| Dr. Negri | 21 | 8 | 1 | 1 | — | 2 | 2 | — | 7 | Do. |
| Mr E Nettlefold | 2 | — | — | 2 | — | — | — | — | — | Do. Case xii. xiii. |
| Dr. Ryan | 2 | 1 | — | — | — | — | — | 1 | not sta'd | Do. Case xiv. xv. |
| Totals | 78 | 37 | 5 | 12 | 2 | 4 | 2 | 1 | 5 | |

‡ Employed the Secale in Hæmorrhages for the last two years "with invariable success."

† We mean those who have used it successfully in this disease, but the number was not stated.

We seldom ordered it in more than five or six grain doses, more or less frequently repeated according to the violence of the case, or the peculiarity of the concomitant symptoms. We *purposely avoided* giving the secale intermixt with other medicine; but we were obliged sometimes to modify by other means the morbid condition of those parts or organs over which our remedy had to exert its powerful action; when at other times it was necessary to get clear of these irritating causes, which would have counteracted its beneficial influence—as, for example, the employment of purgatives when the bowels were costive. This, however, can never be an objection to our practice, as that *must* be always the case with the administration of any other remedies, which are given with a peculiar object. Although the criterion of the *post hoc, ergo propter hoc*, be not always correct, still we believe that in our profession, when violent symptoms are present, and we employ remedies of acknowledged activity, with the view of curing them, if we obtain a favorable and constant result for a sufficient number of times, we may begin to believe that criterion sufficiently correct. The weight of such a conclusion is moreover increased by the uniformity of results obtained by different individuals, and in different countries, therefore the following prospective view of the general results of the secale cornutum in hæmorrhages and leucorrhœa, will make a striking impression of the real efficacy of that remedy against those classes of diseases.—*London Med. and Sur. Journal—From Balt. Medical and Surgical Journal and Review.*

4. *Herpes.*—In a case of herpes in a female, at present in the Westminster Hospital, Mr. Guthrie has employed the acetate of copper, as an external application, with very decided success. This ointment was originally employed by an old women, who, about thirty years ago, undertook to cure some very severe cases of herpes at that time in the hospital. Her treatment was completely successful, but she refused to divulge the nature of the ointment. It was, however, analyzed, and found to be composed of acetate of copper. Ever since that period the acetate of copper has been applied in like cases with uniform success.—*London Med. and Sur. Jour.—Balt. Med. and Surg. Jour.*

5. *Fresh Prepared Carbonate of Iron.*—Doctor Bûchner and Dr. Richter both attest the efficacy of this mineral salt, even in cases of neuralgia, where common, not recently prepared, carbonate of iron had failed. The mode of administering the remedy is as follows—twelve grains of crystalized sulphate of iron are to be mixed with six grains of dried carbonate of soda, and dissolved in half a cupful of water, slightly sweetened with sugar: this dose to be taken three times a day. It is equivalent to five grains of carbonate of iron, and operates as an anti-neuralgic medicine, much more powerfully than larger quantities of the carbonate of iron as sold in the shops.—*Dublin Journal, Jan.* 1834.—*Balt. Med. and Sur. Jour.*

6. *Ranque's Remedy for Swollen Breasts.*—Every thing which can add to our knowledge concerning the best means to be adopted in cases where the mammary glands become swollen, painful and indurated, in consequence of the child being taken from the breast, and the discharge of the milk by the natural outlet ceasing, must be thankfully received. Doctor Schnur has made an interesting communication on this subject. He was struck by some observations made by Ranque, (Floriep's Notizen,) and determined to try the remedy he recommended.

The swelling of the breast which precedes the formation of mammary abscess, is caused in the first instance by the retention of the milk and the consequent distention of the lactiferous ducts. But this is not the only cause of the local derangement that so speedily follows, for the vascular system of the mammæ is wonderfully increased preparatory to and during lactation, and, therefore, when this augmented circulation of the breasts is baffled in the performance of its proper function, the secretion of milk, it often tends to form with great rapidity vicarious and unhealthy products. Hence arises the obstinacy of many such cases, and hence they are frequently not found to be amenable to the common methods of treating local congestions or inflammations. All practical men are consequently obliged to adopt various methods of treatment, and the skillful accoucheur is often enabled by attention and pains to save his patient from the suffering accompanying such affections. Ranque, impressed with certain theoretical ideas which is unnecessary here to discuss, was led to the use of the following liniment:—

℞. Extracti Belladonnæ ℈ ii.

Aquæ Laurocerasi ℥ ii.

Ætheris Sulphurici ℥ i.

Ft. Linimentum.

This must be well shaken before it is used. It is to be rubbed into the breasts as high as the axillæ, morning and evening, and the breast must be then covered with fine flannel soaked in the liniment. This proceeding must be repeated every day, until the swelling disappears, which is usually on the second or third day. The æther has a smell which to some is very disagreeable, but they ought to bear this inconvenience if possible, for it adds essentially to the efficacy of the remedy.—*Dublin Jour. Jan.* 1834.—*Balt. Med. and Sur. Jour.*

7. *Facial Hemiplegia—External use of Phosphorus.*—The symptoms of this local palsy are well known; the mouth is drawn to the sound side, the eye is half-closed and weeping; the point of the nose is sometimes distorted, and the patient is often utterly incapable of moving the forehead, eyelids, and nostrils of the affected side; the motion of the eyeball, however, remains perfect; and the saliva usually flows more profusely than in health; but part of the food, especially if it be liquid, is apt to escape from one corner of the mouth. The temperature of the palsied parts is often lower than that of the other half of the face. The general health may be quite unimpaired. This hemiprosopoplegia may happen at any period of life; but in childhood it is very rare. The following treatment was successful in three cases.

℞. Phosphori, gr. vj.

Olei animalis æther, ℥ iij. M.

The palsied parts are to be rubbed with this embrocation three or four times daily. After it has been used for a day or two, several places become sore, and then form scabs or crusts, which gradually dry and fall off. The rubbing must be renewed a second time, when the skin recovers its soundness; and in severe cases the operation requires a third repetition. Generally after the first desication, the parts are found to have regained a slight power of motion, which increases more and more after the second and third rubbings. The use of the liniment causes very considerable pain, and a feeling of burning; but no evil effect has ever resulted from it.—*Hufeland's Jour.—Medico-Chirurgical Review, Jan.* 1834.—*Balt. Med. and Sur. Jour.*

8. *Radical cure of Varicocele.*—In a memoir recently read by M. Breschet before the *Academie des Sciences*, a new and effectual method is proposed for the radical cure of cirsocele and varicocele. M. Breschet had, for a length of time, reflected upon the practicability of accomplishing this object by effecting the complete obliteration of the varicose veins, and an opportunity presenting, he resolved to put it to the test. With this view he had constructed small steel forceps, so light as not to incommode by their weight, and arranged in such a manner as to admit of the pressure being regulated by a screw. To obviate any material injury of the skin, the bite of the instrument was made somewhat broad and flat, and was besides carefully padded. In the application of these forceps, the skin was pinced up with the varicose veins, while the vas deferens was left in the posterior part of the cord. One pair of forceps was then applied to the fold of the skin, and the varicose veins thus pinched up, as near to the abdominal ring as possible, and was fixed there by turning the screw, so as to make sufficient pressure to prevent the flow of blood through the veins. A second was afterwards applied, in the same manner, in the immediate vicinity of the testicle. To secure the equable operation of the constriction, meshes of charpie were interposed between the blades of the instrument and the skin. In one case in which this practice was pursued, the individual complained towards evening of a slight sense of heat in the part, and pain extending upwards to the lumbar region. He was put upon rigid diet, and was directed to keep the part constantly wet with a solution of the acetate of lead. These applications allayed the pain, and the next day it was so much reduced as to be supportable. The part of the tumor situated between the two forceps was somewhat red, hot and tumefied, but the portion included in the bite of the instrument was not more painful than other points.

At the expiration of forty-eight hours, sufficient inflammation having been excited to effect the adhesion and obliteration of the veins, the instruments were removed, and the inflammation of the parts was treated by resolvents. The compression exercised upon the skin gave rise to a small eschar, followed by a superficial ulcer, which healed in about eight days. The varicose tumor, which was at first red and tumid, gradually wasted away, and at the expiration of a fortnight, was reduced to the size of a small nut, and when pinched between the fingers, none of the venous cords could be distinguished, but the whole seemed to be converted into a simple homogeneous mass. A complete cure was accomplished in this case in about twenty days, at the expiration of which time the diseased cord was not larger than that of the opposite side. M. Breschet also reports another case, which was successfully treated by the same procedure.—*Gazette Medicale, Jan.* 1834—*Balt. Med. and Sur. Jour.*

9. *Case of Hypertrophy of the Muscular Coat of the Stomach.*—Dr. Otto, of Annaberg in Saxony, relates in *Hufeland's Journal*, for February, 1833, an interesting case of hypertrophy of the muscular coat of the stomach, a pathological condition of rare occurrence. Morgagni, Haller, Baillie, do not mention it. Meckel, in his Pathological Anatomy, only observes in a general manner, that the parietes, especially the muscular coat of the stomach of great eaters, are thick. Beclard in his additions to Bichat's General Anatomy, does not notice this kind of hypertrophy, and speaks only of that of the heart and bladder. Louis has noticed this subject more particularly than any other French writer, in his Anatomico-pathological Researches.—When treating of cancer of the pylorus, he quotes two cases of hypertrophy of the muscular coat of the stomach, which he compares to the parietes of the ventricles of the heart, the fasciculi were prominent; in both cases there was scirrhus of the pylorus, and M. Louis proposed the question, whether the contraction of the duodenal orifice of the stomach was the cause of the hypertrophy. In the following case there was neither schirrus nor contraction of the pylorus, and the opinion of M. Louis that these two diseases are independant on account of the frequency of the one and the rarity of the other, is confirmed.

A woman, forty-nine years of age, thin, sanguineous temperament, mother of several children, had experienced many domestic troubles, from which she endeavored to distract her mind, by indulging her appetite for eating, which became incredibly voracious. In the summer of 1827, she complained of periodical dyspnœa, and swelling of the right foot; her menses ceased; she became emaciated; her appearance was that of a person in bad health. Nevertheless, she had no tumefaction of the abdomen, nor pain there on pressure; her appetite was good: evacuations regular; tongue clean, and she never complained of nausea, borborygmy, hiccup, or laborious digestion. The respiration was easy, pulsation of the heart feeble, the pulse normal. She was remarkably depressed in spirits. The predominant symptom consisted in a periodical dyspnœa, which recurred every evening, sometimes even during the night, and produced a sensation of weight in the lower part of the abdomen, which rose up like a ball towards the heart, and impeded her breathing. These symptoms continued for six or eight minutes, and always a long time subsequent to a meal. Their intensity became so great, that the patient was at times delirious; she even at one time attempted to hang herself. At the commencement of winter the emaciation made alarming progress, withuot the appetite being diminished, or the digestion deranged, and although the patient was so enfeebled, that she could not quit her bed. The violence of the paroxysms constantly increased; the marasmus took place, with subsultus tendinum, weakness of the organs of sense, coma, loss of sensibility, delirium, and finally, death on the 19th of December, 1827.

*Autopsy, ten hours after death.*—The pectora organs were healthy; the heart small; the pericardium contained a little serosity; omentum wanting; liver and spleen normal; stomach shriveled and thickened, especially towards the pyloric region and along the larger curvature, which appeared less extended than the smaller, its blood vessels empty; its mucous membrane thin, almost translucent, ordinary consistence, covered with a viscid and thick mucus, elevated by numerous muscular fasciculi, the size of a goosequill, the direction of which was from the great cul-de-sac to the pylorus. These gave to the internal face of the stomach the appearance of that of the heart. Beneath these fasciculi the muscular coat formed a layer of half an inch in thickness. The remainder of the intestinal canal was in a normal state, as were also the urinary passages. This case is interesting in a double point of view; 1, in a pathological aspect, connecting the stomach with the other muscular sacs; 2, by the almost total absence of symptoms referrible to the digestive organs.—*Rev. Med. Aug.* 1833.—*Am. Journal of the Medical Sciences.*

10. *Remarks on Local Diseases.—Pathology of the Diseases of the Digestive System.* By William Stokes, M. D. Extracted from a lecture on the Theory and Practice of Medicine, delivered at the Medical School, Park street, Dublin. Session 1833–4.—Gentlemen, I commence with the digestive system. I am anxious to do this for several reasons, but for none more than this—that to the improvements made in the pathology of the digestive system we owe much of the rapid advancement of modern practical medicine. Before our time the pathology of the digestive system was very little known, and if not quite a *terra incognita* in medicine, there existed respecting it a great deal of misconception. The schools were deeply tinctured with the doctrines of the Humoralists and the Brownists; and this had the effect of giving rise to irrational theories and false notions of the true state of the system in disease. The humoral pathologists, who sought for disease in an alteration of the fluids alone, neglected the study of visceral lesions; and when they turned their attention to the digestive system, they only considered it, its secretions, and not its actual condition or the state of its sympathies. The liver, with them, was an organ of the highest importance, and the secretion of bile claimed a vast share of their attention. To it they gave a paramount influence, and to an alteration in its quantity and quality they attributed most of the changes which occur, not only in the digestive tube, but also in the whole system; and hence the great object of their practice was to attempt to restore its healthy condition, convinced that if this were once accomplished, every thing would go on favorably. From this too, arose the purgative plan of treatment in various forms of intestinal disease, a plan too often rashly pursued, even where there was unequivocal proof of inflammation in the digestive tube. Their sole purpose was to evacuate sordes, to produce a flow of healthy bile, and to eliminate depraved secretions; and they did this without possessing any knowledge of local inflammation, or of the effects of disease of the digestive system on other organs. The followers of Brown, on the other hand, only admitted disease of the digestive system in a state of intense, manifest violence, as for instance, ileus, or violent enteritis; but in the great majority of cases, they did not recognize intestinal inflammation, because their prominent symptom was prostration, or to use their own terms, an asthenic condition of the whole system. They saw nothing but prostration; they prescribed for nothing but debility; they gave wine instead of iced water; ordered bark instead of local depletion. They exasperated the disease by stimulants; and then, thinking they had not gone far enough, they heightened the stimulant and doubled the debility.

Another cause of the low state of pathology in former times was the general neglect of dissection. The fact is, that in fever there were no post mortem examination made, until very lately. Morgagni, who did so much for pathological anatomy on almost every other subject, did litttle for fever, because he was afraid to dissect the bodies of persons who had died of a contagious disease. This was the idea which prevailed among the older pathologists; and hence this source of knowledge was avoided, and for many successive centuries the state of the viscera in fever was a matter of speculation, doubt, and uncertainty. Even at the present day it is only done by the ardent pathologist, who cares not about filth and stench, and who had rather encounter the miasm of contagion than remain in the mists of error. Nothing is more common, I regret to say, even at the present time, than this:—A person says he has dissected cases of fever, and when asked whether he had examined the intestinal canal, he says that the intestines appeared healthy, but he did not make any particular inspection of them; he only opened the belly, and finding no trace of inflammation in the peritoneum, he went no further. Now nothing can be more useless than such an examination. If we compare the information afforded by an inspection of the serous membranes of the three great cavities, we shall find that the least is given by an examination of that of the abdomen. Disease of the substance of the brain is rare without affections of its investing membrane, disease of the substance of the lung is exceedingly rare without the occurrence of the disease of the pleura, but you may have most extensive and fatal disease of the intestinal canal without the slightest lesion of the peritoneum. In this point, therefore, it differs from the pleura, and from the arachnoid membrane. The fact of the rarity of disease of the peritoneum in cases of disease affecting the parts beneath, was noticed by Dr. Graves and myself, in our report of the Meath Hospital, and also by Mr. Annesley, in his account of the diseases of India. You will see cases of hepatic abscess, which present a distinct tumor externally, and where you can detect a perceptible fluctuation; and yet, if you examine these cases after death, you may not find any adhesions of the peritoneum, even in the situation of the abscess. You will find the mucous and muscular coats of the colon extensively destroyed, you will see the stomach all but perforated, you will meet with cases where the whole ileum is one extensive sheet of ulcerations, with no disease in the adjacent peritoneum.

In entering on the consideration of diseases of the digestive system, we shall begin first with the mucous expansion of the stomach and intestines, and then proceed to the affections of the solid viscera connected with them. The mucous surface of the stomach and intestines is of enormous extent and extraordinary sensibility, possessed of innumerable and powerful sympathies; its influence is felt by almost every organ in the body, formed for receiving and elaborating every thing destined for nutrition; its conditions, both in health and disease, are entitled to the deepest and most attentive consideration. To facilitate the study of its affections, and for the sake of some practical arrangement, we shall divide its diseases into five classes, beginning with the œsophagus, or that portion of the digestive tube which is above the diaphragm, and then proceeding to the stomach, duodenum, ileum, colon, and rectum. But in order to give you a clear idea of diseases of the intestinal canal, I shall commence with diseases of the stomach; because, if you consider the whole range of animal life, you will find that its functions are the most important, the stomach constituting, as it were, the source and fountain of life, which is nutrition, and giving by its existence a character to all the upper classes of animals. No organ possesses such remarkable sympathies as the stomach, whether we look upon them as sympathies of organic or animal life, none possesses such remarkable power and influence in modifying the conditions of every part of the system. But, putting aside physiological reasons, let us come to practical matters. The success of almost every form of medical treatment, all the advantages to be derived from the administration of internal medicine depend upon the stomach; in fact, in whatever point of view we consider it, we must look upon a knowledge of the state of the stomach as the great key to sound and successful practice.

It is a most useful reflection to consider the extraordinary frequency of disease in some portion of the digestive tube. It is now admitted by every person possessed of experience in the causes of mortality, that more human beings die with acute or chronic diseases of the digestive tube than with diseases of any other part of the system. This has been established by numerous investigations, and is admitted by the best pathologists; and, indeed, I think it can be easily accounted for, when we call to mind how many persons die of some form of fever or other, when we look to the ravages of remittent and yellow fever, to the hundreds of thousands who annually perish by the various classes of fevers. Now in almost every one of these cases, disease of the digestive system forms one of the most prominent pathological characters. Recollect, besides, all that die of dysentery, whether sporadic or simple, and here is inflammation of the colon; see too, how many die of diarrhœa,—here too, there is intestinal disease; remember how many die of the malignant intermittent of the West Indies, in which unequivocal proofs of disease of the stomach and intestines have been found. Observe what a close connexion there is between tabes mesenterica, and inflammation of the mucous membrane and surface of the intestines; think what a vast number of persons fall victims to the harassing effects of constipation and dyspepsia; and recollect there is a host of diseases in which the train of morbid phenomena commences in the digestive system, and then exhibits itself by functional alteration or organic diseases of other parts.

Gentlemen, we recognize the presence of disease in the digestive tube, first by the local phenomena and the lesion of the digestive function, and next by the sympathetic relations of other parts, by the sympathies of the respiratory system, of the circulation of the skin and of the nervous system. I shall enumerate the local phenomena and functional lesions: vomiting, anorexia, thirst, jaundice, pain, tenderness on pressure, tympanitis, changes in the character and quality of the discharges, constipation. Here are a set of functional lesions and local phenomena, let us now consider the sympathetic relations; these are fever, heat of the skin, suppression of the cutaneous secretion, suppression of the secretion of urine, morbid states of the tongue and pulse, pains in the chest and cough, hurried breathing, and palpitations of the heart. In the next place, we may have prostration of strength, delirium, coma, convulsions, tetanic spasms, and other symptoms of functional diseases of the brain; these are all sympathies of relation. Now, in the first place, I have to remark, that there is a great deal of variety in the combination of these symptoms. On what does this depend? on a variety of circumstances; sometimes on the intensity or extent of the inflammation; sometimes on the situation of the disease; sometimes on the complication of the affection; sometimes on the various modes and degrees of susceptibility of the individual. All these causes tend to produce a great variety in the disease, and an extensive modification of the sympathetic relations. For instance, in some cases, inflammation of the stomach and intestines is so slight that the patient is not prevented from going about and pursuing his ordinary avocations; in others, on the contrary, the patients are struck down at once by the violence of the disease, and are carried off by the fever which accompanies it before the inflammation is completely developed. It varies also according to situation; there is a difference between gastritis and dysentery, in the former we have an inactive state of the great intestine, and consequent constipation in the latter, the colon is thrown into violent action, and there are frequent dejections. Disease of the duodenum is attended with a very remarkable peculiarity, being very frequently complicated with jaundice; here is a modification produced by situation. Again, inflammation of the ileum is attended with a very curious peculiarity, namely, the absence of pain. The patient states, that he feels unwell, he has obscure symptoms of intestinal disease, but it is neither dysentery nor gastritis; you investigate it with care and find that the ileum is in a state of inflammation. Yet the patient does not complain of any pain, and this is another peculiarity depending on situation.

But in considering the differences which depend upon intensity, extent, and situation of disease of the intestinal canal, we must not omit those which depend upon tissue. If disease be confined to the mucous membrane of the intestines alone, we may have an extremely diffused and extensive inflammation sufficient to destroy life, without any pain being complained of by the patient, it is a painless though fatal disease. Recollect this, extensive and fatal inflammation without pain. In former times the ideas of pain and inflammation were inseparable. Thanks to the light which pathology has shed upon modern medical science we are now acquainted with this seeming anomaly, and can conceive the existence of extensive disease of mucous surfaces unaccompanied by pain. But let the inflammation seize on the muscular tissue, the character of the disease is instantly changed, and the pain is dreadful. Here is a case in which difference of tissue is to be taken into consideration.

The phenomena and sympathetic relations of intestinal disease may vary also according to its complication, and here we come to investigate one of the most beautiful laws of the human economy, namely that the more complicated a disease is the more latent will be any local, lesion. This is a point that should never be forgotten. For instance, enteritis by itself is much more easily recognized than when complicated with pneumonia, or with irritation of the brain, and gastritis is but too often completely masked by being combined with irritation of the bronchial mucous membrane. Lastly, we have the varieties which depend on different degrees of susceptibility. In one person we have only slight cerebral irritation, in another high excitement, in a third delirium and extraordinary convulsions. The variety, then, in the modifications of disease, and the combinations of sympathies is very great, and is referable to the extent and intensity of the inflammation, difference of situation, complication of disease, difference of tissue, and different degrees of susceptibility. I shall give examples of these at my next lecture, and then proceed to the pathology and treatment of gastritis.—*London Med. and Surg. Journal, Jan.* 4, 1834. —*Am. Journal of the Med. Sciences.*

11. *Ointment for the cure of Porrigo.*—M. Biett considers the following as one of the most powerful agents for the cure of porrigo. ℞. Ioduret. sulph, ℈j. to ʒss.; axung. ℥j. M. A drachm is usually employed at each friction.—*Bulletin Gen. de Therapeutique, Tom. IV.*, 1833. —*Am. Jour. of the Med. Sciences.*

12. M. Aubergier's *Pomatum for Preventing the Hair from Falling Out.*—℞. Prepared marrow of beef, ʒvj.; sweet almond oil, ʒij.; red Peruvian bark, ʒj. The powdered bark is to be moistened with a small portion of the almond oil, and the remainder then added; when the mixture is made, the marrow is to be melted at a moderate heat, and gradually added to the preceding ingredients in a mortar, and rubbed together until cold. It is asserted to have never failed in preserving the hair.—*Bulletin Gen. de Therapeutique—Am. Jour. Med. Sciences.*

13. *Improved Method of Administering Epsom Salts.*—Dr. James Henry, of Dublin, recommends the sulphate of magnesia given according to the following formula as an agreeable, safe and efficacious purgative. Saturate any quantity of cold water with sulphate of magnesia; filter through paper, and add to every seven ounces of the solution one ounce of the *dilute* sulphuric acid of the Dublin or Edinburgh pharmacopœias.

*Dose.*—One table-spoonful in a wine glass of water.*

In those cases in which the bowels are easily moved, a single table-spoonful is sufficient to produce a considerable purgative effect.

In ordinary cases, a table-spoonful taken an hour or two before breakfast produces one or two evacuations immediately after breakfast.

In other cases, the dose is to be repeated once or twice, at intervals of two or three hours, according to circumstances.

Where the symptoms are urgent, a table-spoonful may be given every hour until the effect is produced; and where the urgency is extreme, a saturated solution of the salt, containing only one-half of the above-mentioned quantity of acid, may be given in doses of two table spoonfuls, repeated every hour.

This combination of sulphate of magnesia and sulphuric acid, administered according to the preceding directions, possesses the following properties.

1. It is an effectual purgative, never failing to move the bowels in all cases in which the bowels can be moved by medicine. I am not acquainted with any purgative which is more certainly effectual.

2. It is quick in its operations; the effect being produced in ordinary cases within two or three hours after the first or second dose, and a necessity rarely arising for the continuance of the medicine beyond the third dose.

3. It is safe, never purging so as to produce exhaustion.

4. It does not give rise to the slightest degree of nausea, but, on the contrary,

5. Quickly puts a stop to nausea, and appeases irritability of the stomach.

6. Flatulence, that most distressing attendant upon constipated bowels, is immediately and signally relieved by this medicine, which not only promotes the expulsion of the *flatus* already generated, but diminishes the tendency to its further secretion.

7. In a few minutes after this medicine has been swallowed, so agreeable a sensation of warmth is felt in the stomach, that the medicine is not only readily taken, but even relished by many persons whose stomach will not retain any other liquid purgative, unless impregnated with the hottest aromatic tinctures.

8. The operation of this medicine is not attended by either sickness, faintishness, or griping. In this respect the acid saline solution possesses a remarkable superiority over all the purgatives in common use.

9. This medicine can be taken every day, or every second day, for a considerable length of time, not only without impairing the stomach

* Each table-spoonful contains about two drachms of sulphate of magnesia, and half a drachm of dilute sulphuric acid.

or other digestive organs, but with manifest advantage to them.*

10. The continued use of this medicine does not produce that irritation of the *rectum* which so commonly attends the continued use of other purgatives.

11. This medicine is not disagreeable to the sight, being perfectly limpid and transparent as the purest spring water.

12. It has no smell.

13. The bitter nauseous flavor of the sulphate of magnesia being almost completely hidden by the acid, the taste of the solution can hardly be said to be at all disagreeable, and it is certainly much less so than that of most other purgative liquid purgatives.

14. It is cheap.

15. It is easily procured every where.

16. It keeps for an unlimited length of time.

From the experience of three years, during which I have made daily use of this purgative in the course of my practice as a physician, I have ascertained that the acid saline solution possesses the properties which I have just enumerated.—*Edin. Med. and Surg. Journal, Jan.* 1834.—*Am. Journal of the Medical Sciences.*

14. *Iodine in Mercurial Salivation.*—In our last No. we stated that iodine had been successfully employed in Germany as a cure for mercurial salivation. We are now enabled to furnish some further details as given by Dr. Kluge, of Berlin, in *Hecker's Medical Journal.*

"Professor Knod Von Helmenstreitt, in Aschauffenburg, was the first who recommended *iodine in mercurial salivation.* (See Hufelan's Journal, May, 1832.) As the syphilitic wards of the great hospital, (Charité) in Berlin, afford numerous examples of this affection, I determined to give iodine a fair trial, and for this purpose I selected seventeen cases, viz. twelve women and five men, all of whom labored under severe mercurial salivation. Helmenstreitt's first directions were to dissolve five grains of iodine in two drachms of spirit of wine, to which two ounces of cinnamon water, and half an ounce of syrup are to be added. Of this the patient was to take at first half a tablespoonful four times a day, which dose was to be gradually augmented two, four, six or even eight grains daily. His latter directions prescribed two grains, or even more, the first day, which dose was to be rapidly increased.

"Two young women who lost four or five pints of saliva daily, were cured in three days by eight grains. One man and one woman got well in four days, having taken ten grains. In two men and four women the ptyalism ceased entirely in six days, during which each had taken from twelve to sixteen grains. In two men and two women the spitting was cured on the seventh day after iodine had been taken to the amount of from twenty to twenty-eight grains. In the latter cases

* The frequently repeated contact of the acid saline solution being injurious to the teeth, is is useful to adopt the precaution of taking the medicine through a quill, or from the spout of a small tea-pot, whenever it is necessary to continue its use for any length of time.

it was, however, remarked, that the great pain of mouth and fœtor of the breath were notably diminished after one day's use of the iodine. In two young women the remedy appeared at first to be of little or no use; in both the salivation amounted to three or four pints daily at the termination of the seventh day, and the only advantage gained appeared to be a certain diminution of soreness of mouth. One of these patients was then obliged to desist from the use of the remedy on account of some constitutional symptoms, and I looked upon this case as a failure. This conclusion was however too hastily made, for the good effects of the iodine began to appear on the following day, and on the third day after she had left it off, that is, on the eleventh from the date of its first exhibition, all morbid secretion of saliva had disappeared, and the gums had very nearly recovered their healthy appearance; in short the patient had recovered, having consumed thirty-four grains of iodine. In the other young woman we stopped the exhibition of iodine on the tenth day, at which time she had taken thirty-six grains, with the effect of diminishing the daily secretion of saliva from five to three pints On the twelfth day she was well.

"In one girl the accidental occurrence of erysipelas of the face prevented the continuance of the remedy. The use of the iodine did not produce in any one of the patients any disagreeable or untoward symptoms, and as I kept them all for some time in hospital after the salivation had ceased, I have the pleasure of likewise testifying that the cure was not only safe, but permanent."

Dr. Graves of Dublin, has also tried the effects of iodine in arresting salivation, with favorable results.—*Dublin Journal of Med. and Chem. Sciences, Jan.* 1834.—*Am. Jour. of Med. Sciences.*

15 *Remarks on the Nature of Neuralgias, and their Treatment.*—By M. Piorry.—As the vast majority of neuralgic affections do not terminate fatally, the lesions which they may produce in the nervous tissue, remain as yet almost unknown; and it is impossible for us to decide whether an irritation, or even indeed a hyperemia has, or has not existed. The larger proportion of cases of pharyngitis treated at the hospitals Saltpétriere and La Pitié, though exhibiting a short time before death all the symptoms of this affection, when examined after death, offered no traces whatever of the disease. If such is the case in inflammations of the mucous membranes, may we not with more reason attribute to the affections of the nervous system a similar series of phenomena. The morbid lesion which attacks the nervous trunk, may in like manner affect separately and independantly the numerous filaments which enter into its composition, and which are so exceedingly minute, that the anatomist labors in vain to isolate them. How is it possible then, for us to decide upon the different shades or tints which these filaments may assume. This circumstance is deserving of recollection, for it is by the color of the nervous matter of the brain, that the pathologist pronounces upon the previous existence of inflammation in this organ. The blood-vessels supplying these nervous filaments are also so exceedingly minute, that to be able to appreciate in them a state of hyperemia, it is necessary that they should be enormously enlarged. Moreover, the cellular tissue of a nerve, which connects its filaments to each other, may take on inflammation, and present a red color, without the filaments themselves being at all concerned. The causes which operate in the production of neuralgic affections, are the same which produce in other organs irritation and hyperemia. A contusion, pressure, violent and sudden muscular contraction, a carious tooth operating as an irritant to the nerve supplying it, an organic lesion of the heart, an articular rheumatism, which, whatever may be said to the contrary, is in fact an inflammation of the joints; a tumor situated upon a nerve, an active inflammation of the surface of the body, extension of the inflammatory action from the intestines to the nerves supplying them, certain movements of the muscles of the head, acting upon some of the neighboring nerves, extension of the irritation which accompanies cancer, and the pressure which an enlarged gland sometimes produces upon a nerve, are some of the appreciable causes which tend to produce these affections; and although we are unable to seize upon these, or similar ones in all cases, yet we should not conclude on this account, that they were not present, but rather ascribe to the imperfections of our means of investigation, their escape from our senses! The group of symptoms does not distinguish neuralgia from neuritis, only the one is transitory in its effects, whilst the other remains stationary for some time. This is to be attributed to the morbid action resting in the first case at its first or mildest stage, whereas, in the second, it passes on to its second, or most violent degree. Perhaps the one may be considered as bearing the same relation to the other, that a cerebral congestion does to a softening of the brain. This comparison, it is true, does not throw much light upon the nature of the case, for we have yet to form positive opinions relative to cerebral congestions and cerebral softenings; nevertheless, the analogy between the two affections is so strong, that it may be well to make use of the simile. Because the pain is not so augmented by pressure, because it assumes various characters, because it may be subject to remissions, and because there is neither redness, heat, nor swelling, nor indeed any of the precursors of inflammatory action present, we must not conclude, that no congestion or inflammation of the nerve exists; for pressure does not always produce pain in cases of congestion or inflammation in other parts. Moreover, the hyperemias which succeed to a lesion of a nerve, or which may accompany it, are subject both to variations in their nature, as well as to remissions. Again, from the nerve being invisible, it is of course impossible to observe the different changes in color, the degree of heat, or of swelling which it may undergo; and finally, there are many cases of inflammation, in which none of the precursory or pathognomonic symptoms of this condition show themselves. M. Andral has well observed, that the symptoms given by authors as characterizing these affections, may lead us to mistake a neuralgia for a neuritis, and vice versa. He considers the best means of distinguishing the two affections from each other, to be the augmentation in volume of the nerve affected with neuritis, but unfortunately in the great majority of cases this augmentation cannot be detected. The results of different methods of treatment do not establish more definitely the differences that exist between the two diseases, for it has happened, that in cases which were essentially neuralgias, sanguine evacuations either produced an amelioration of the symptoms, or cured them completely, or changed them from continued to intermittent, and this too as speedily and as effectually as they did in cases which were considered as well marked neutritis. On the other hand, the sulphate of quinine operated very beneficially in a case, the intermittent symptoms of which seemed to be dependant upon neuritis, which had originally been accompanied by a physical lesion of the nerve, (a neuroma.) It most frequently proves useful in those cases in which the previous application of leeches had produced decided relief. Hence, even admitting theoretically, that the neuralgic affections constitute two degrees in the lesion of a nerve, or even that they are essentially different in their nature, it must be confessed, that neither the pathological phenomenon, nor indeed the treatment, furnish signs sufficiently well-marked to induce us to form very different therapeutic indications for these two morbid conditions.

The same remarks apply to the efforts which have been made to establish a diagnosis between neuromyelitis and neurilemmitis. The observation of Riel has been copied by every one, without its truth having ever been properly ascertained; it would have been much better to have tested its truth by repeating his experiment, and until it is positively shown that the neurilema of a nervous filament may take on a state of inflammation, whilst the pulp it contains remains unharmed, and vice versa, we cannot give credit to the subtle divisions made of this class of diseases, which divisions were certainly never deduced from sound pathological investigation. The neuromas mentioned by Galen, Valsalva, and Petit, and upon which M. M. Dupuytren, Alexander, Boisseau, Beclard, Descot, Andral, Schiffner, and Cruveilhier, have published such important and interesting observations, seem in some cases to be dependent upon the previous existence of some cancerous affection, and in others to result from the morbid action which constitutes neuralgia and neuritis, which affections in turn make their appearance. Their symptoms are the same, though the treatment is different, for in three cases we know positively the situation of the organic lesion, and also that where other remedies have failed, an operation is absolutely required to affect a cure. There are, however, certain distinctions which may be drawn between the neuralgias and the neurites, according to the nature of the cause producing them, or the peculiar nerve attached. M. Lembert, who has published some very useful observations upon the endermic method of medication, is induced to believe from several facts which he has noticed, that certain nerves, which he thinks are more vascular than others, and which preside over the functions of touch and nutrition, such for instance, as the branches of the fifth pair, are more disposed than the other nerves of the body to attacks of neuritis, andless to attacks of neuralgia, and that sanguine emissions are more useful in the first class than in the second. Experience and future observation upon the functions of the different nerves must decide the merits of this opinion. In all cases where the nerve undergoes a morbid alteration sufficiently violent to give rise to symptoms, a peculiar pain, which is propagated through all the nervous ramifications, and which in some cases seems to proceed from the branches to the trunk, manifests itself. The sensation produced, is that of stiffness or painful numbness, accompanied by a pricking and vibratory feeling, which continues a longer or a shorter period, according to the nature of the circumstances that determine its production. If the exciting causes suddenly cease to operate, and if the lesion sustained by the nerve has been trifling in its extent, the pain gradually subsides, and in a short time every thing reassumes its original healthy action. (The pain caused by pressure of the cubital nerve at the elbow-joint, or of the sciatic nerve where it emerges from the pelvis, or in the popliteal space, and that produced by the pressure of the child's head during labour, may be cited as examples of this kind.) If, however, the exciting cause is more violent in its operation, the painful sensations continue for a longer time, or should they perchance abate or disappear for a time, soon return. The exacerbations take place from time to time, and in some cases obey the law of periodicity, for in disease, as well as in health, the nervous functions possess a tendency towards intermission. (The pain caused by a carious tooth, constituting odontalgia, that produced by cancerous tumours in the aim-pit and uterus, which irritate or compress the nervous trunks of the arm and thigh, &c., may be placed among the examples of this species.) In these cases the pain persists as long as the cause producing it continues to operate, and is incurable unless the latter be removed. The pain which is at first confined to the trunk, or filament affected, may gradually extend itself to the neighbouring nerves, (odontalgia following some of the various neuralgias of the face,) or it may make its appearance in several points of the nervous system at the same time. In these cases the secondary neuralgic symtoms may generally be relieved, but it is not until the primitive affection is recognized and eradicated, that we can trust to their non-appearance again. Consecutively to the operation of either a transitory or persistant cause, the nerve may become the seat of a simple irritation, which cannot be conceived to occur, except in cases of anemia or chlorosis, without a congestion of the sanguine capillaries, since all organs in a state of excitation have their capillaries injected. In this case, however, we are obliged to acknowledge the existence of such a state of things, by analogy alone, since it is impossible to examine the peculiar condition of the nerve during life, and if examined after death it does not present the same characters, which it would have done if observed during the existence of pain. Yet in cases of irridian or ophthalmic neuralgia, as soon as the symptoms declare themselves, the eyelids and the neighbouring parts are found to be in a state of congestion, if the disease is confined to this degree of excitement and primitive congestion, we have only a neuralgia produced. When an attack of neuralgia has existed for any length of time, it frequently happens that there exists great tendency to a relapsé, notwithstanding the original cause of its appearance has been completely eradicated. It seems in these cases that the system acquires a sort of habit, which it is extremely difficult to overcome. If the cause has operated with violence, or is continued for any length of time, or should the disease be augmented a degree in intensity, an inflammation of the nerve may be the result. The neuritis thus brought about may be either followed or accompanied by hypertrophy of the nerve; by a deposition of blood or pus between its filaments; by the formation of hard or scirrhous tumours in its substance; or of small cysts, the parietes of which are hard, and contain a sort of gelatinous fluid; or of encephaloid degenerations, &c. The pain experienced in the nervous trunk, or in its filaments, is so peculiar in its character, that it is impossible to confound it with any other. *It resembles precisely that which is perceived when the inner side of the elbow-joint is suddenly pressed upon or struck.* In doubtful cases of chronic rheumatism or gouty arthritis, or of musculitis, &c., the absence or presence of this peculiar sensation will decide at once the character of the affection, and it is important to bear in mind this fact in the examination of our patient.

*Treatment.*—Reasoning from the facts just given, the indications to be observed, and the course to be pursued in the treatment of neuralgias, seems to be as follows. 1st. Endeavour by all possible means to find out the material or organic cause which has produced, or which is at the time operating in the production of either the neuralgia or neuritis, and destroy it if possible at once.

*Examples.* (Extract the carious tooth in odontalgia dependent upon its irritating properties; prevent certain muscular movements which seem to operate in the production of the disease; remove tumours situated upon the nerve, &c.,) should the organic cause be entirely beyond the reach of our therapeutic agents, (as for instance in cancer of the uterus determining nervous pains,) we must content ourselves with the administration of such palliatives as we may possess. If the disease still persists after the removal of the cause which produced it; or should the cause escape our means of investigation; or the attack be of recent occurrence, or even chronic in robust subjects, and sometimes also in persons of a nervous temperament; it will be well, before resorting to any other method of treatment, to try the effect of antiphlogistic remedies. The extent to which these should be carried, must of course depend upon the quantity of blood the patient can afford to lose; which circumstances may be determined by an examination of his arteries, veins, and capillary circulation, and by the percussion of such organs as are highly vascular, and capable of containing large quantities of blood. General blood-letting which proved so successful in the hands of Cotugno, may occasionally be resorted to; but copious local depletion effected by the application of a large number of leeches along the course of the affected nerve, together with rest and poultices, repeated pro re nata, is the course of treatment usually pursued in this stage. Those who doubt the efficacy of sanguine evacuations in such cases, have either not properly studied their effects, or have seen them employed with too much rashness, or with too great timidity. What have we to fear from blood-letting? Is it the momentary debility which it produces? If the precautions which we have elsewhere indicated are observed, the syncope can never become so excessive as to prove dangerous. (*Vide Procede Oper. de la Percussion, &c.*, p. 249.) Is it consecutive debility? If, in the first instance, we do not establish in the system a dangerous degree of debility, by extracting too large a quantity of blood, is there any reason why we should anticipate the occurrence of a consecutive one? Is it the tediousness of the convalescence? This will only take place when the patient is confined to a vigorous diet. At La Pitie, where sanguine emissions were carried to a great extent, but the patient at the same time properly nourished, the transition from a state of disease to perfect health, was, in the majority of cases, almost immediate. Moreover, the animals used in the experiments on this subject, and from whom large quantities of blood were extracted, speedily regained their usual quantity of this fluid. We should not, therefore, doubt the importance of blood-letting in these cases, but it is true that it must be conducted with prudence, and with a proper knowledge of its effects. In the hands of those familiar with its powers, it can never prove dangerous; it only becomes so when ordered by the inexperienced and rash. If the symptoms are ameliorated by this plan of treatment, it should be continued; but we must recollect, that although sanguine emissions when early prescribed are not generally productive of danger, it is not so with those used at a later date. We should, therefore, be guarded in ordering a repetition of this evacuation. These remedies alone frequently prove sufficient to effect a radical cure. When the antiphlogistic remedies have been tried without success, and where the disease does not manifest a tendency to assume an intermittent type, we may resort to the application of vesicatories along the tract of the nerve, according to the plan of Cotugno, which in his hands was very successful. The blister should be allowed to remain but a short time in one spot, and must then be applied to another in the neighbourhood. The form of the blister is also of some importance. In general it should be long and narrow, so as to apply itself to the part of the nerve. Almost every practitioner has witnessed the success of this remedy. In several instances, however, the blister has failed to produce the anticipated effect; we may here denude the cutis of its cuticle, and resort to the endermic method of medication. The narcotics, for instance, the acetate, and particularly the hydrochlorate of morphia, the stramonium, belladonna, henbane, &c., may all be resorted to. In some cases we have found this method prove exceedingly useful, in others again it has entirely failed. A woman of the Salpétriere had complained for several days of an acute pain in the temple, which seemed to be neuralgic in its character; a blister was ordered to be applied to the part, for the purpose of removing the cuticle, and the denuded cutis to be sprinkled over with a grain of the hydrochlorate of morphia. The day after the application of the blister, the patient expressed himselfas entirely relieved. All the benefit derived was attributed to the application of the opium; it was found afterwards, however, that the opium had not been applied, and that the blister alone had effected the cure. To avoid for the future, attributing to the absorption of some remedy, that which is due to the operation of the blister alone, it would be well to apply the blister in a strip of about two lines breadth, and then place the narcotic upon the denuded surface, which of course will be exceedingly small. When the narcotic, opium, for instance, has failed when applied externally in producing its effects, we may exhibit it internally, either in the shape of an enema, or in a draught. These means, however, though they generally produce a momentary alleviation of the symptoms, rarely effect a radical cure. Should the symptoms appear periodically—whether after the lapse of a day or of several weeks, or of several hours, or even less time; if from the influence of sanguine emissions, the disease changes from a continued, with occasional exacerbations, to an intermittent form, we may resort to the administration of the sulphate of quinine in large doses, as if it were a case of intermittent fever! We must not trust to small doses, but give it in doses of ten, fifteen, and twenty grains; the largest dose being given immediately after the occurrence of the attack. If the succeeding paroxysms should be milder in their character, or the interval between them increased, we may either continue in larger doses the sulphate of quinine, or suspend its employment for several days, in order to be able to resort to it suddenly in very large quantities. In some cases it has seemed to me, that a repetition of the local sanguine emission during the paroxysm has been productive of benefit; and that the sulphate of quinine administered immediately afterwards operated more efficaciously upon the approaching attack. In cases of amenia, where the skin and lips are pale, and in young women whose organs contain but little blood, the sub-carbonate of iron sometimes produces the most happy effects, particularly in cases accompanied with irregular menstruation, and where the menstrual blood is paler than usual. Here we must be very cautious how we prescribe sanguine depletion. As the brain, in cases of syncope and anemia, frequently exhibits symptoms precisely similar to those produced by a congestion of its substance, in like manner may the nerves, in cases where there exists a deficiency of blood in the system, should they perchance become irritated, be accompanied by all the symptoms of irritation with congestion. When all our means have failed, and the treatment founded upon rational views of the disease proved unsuccessful, we might then have recourse to empirical remedies. The best to commence with are those which are supposed to operate with most safety to the patient. The pills of Meglin have sometimes proved useful; the essential oil of turpentine has also produced beneficial effects in the practice of M. Martinet. Finally, cauterization of the nerve, as practised by Andre, or its section which Galen is said to have resorted to, and which Nuck so highly recommends, and which in the hands of MM. Maréchal, Louis, Pouteau, Guerin and Delpeck, has produced such varied effects, may be tried. Several very interesting observations upon this latter method of treatment, as well as many important facts in the history of the neuralgia's, will be found in a very remarkable and exceedingly erudite memoir, that has just been published by M. Halliday.—*Gaz. Med. de paris, February 2d*, 1833. —*Am. Jour. Med. Sciences.*

15. *On Sanguineous Tumours of the Cranium.*—The most common and least dangerous sort of these bloody swellings is when the blood is effused between the aponeurosis of the occipito-frontalis muscle, and the common integuments.—They are very often observed on the heads of new-born infants, and are no doubt caused by the severe contution of the cranium, during its expulsion through the pelvis. This is the "caput succedaneum" of some German authors. In general, it may be easily discussed under the use of resolvent applications.

The second variety of bloody tumours of the scalp, and which is usally caused by contution or other external violence, is that which has been described by M. Zeller under the name of cephalæmatomié. The blood is diffused between the aponeurosis and pericranium. The German and Italian writers have often confounded this variety with the former;—it is only on this supposition, that we can account for their differences of opinion with respect to the danger or not of these bloody swellings, and to the treatment which they have recommended; some advising the knife to be used, others trusting to discutient lotions.

The fluctuation is not so distinct as the first-mentioned kind, and the blood becomes diffused more readily, so that it does not generally present the appearance of a depression in the centre, and an elevated hardened border round; signs which have sometimes led surgeons to suppose that there was a depressed fracture of the bone, when the effects of the bruise were nothing but an ecchymosed subcutaneous swelling. In this sort the aponeurosis sometimes form a solid cyst round the extravasated blood. Whenever the pericranium becomes detached from the skull the injury assumes a more grave importance;—we cannot with certainty predict that the bone may not become ultimately necrosed. But this is rare, and authors have no doubt often committed the error of supposing that the blood was in contact with the bones, when the investing membrane of the latter was quite entire and firmly adhering.

M. Velpeau mentions a case of a child, only ten days old, being brought to him for a supposed hernia of the brain.

A soft fluctuating tumour covered the greater part of the left parietal, part of the temporal, and almost the whole of the occipital bone. The dispersion of this swelling was easily effected in the course of a few days. It is quite an unusual occurrence, that the pericranium is detached from the bone in new-born infants, however difficult the delivery may have been, and however large the quantity of blood effused. Sometimes, indeed, when a true encephalocele does exist, we meet with bloody swellings, which have their seat next to the bone, on others parts of the head;—such cases are very generally fatal.

The third species of swelling is situated deeper than either of the preceding two. Chelius, in his manual of Surgery, published in 1827

at Heidelberg, places it in the diploe of the bones; M. Velpeau thinks that it more frequently begins between the bone and the dura mata, although a case mentioned to him by M. Lauth is more favourable to the other opinion. A man received a blow with a cudgel on the parietal bone; but little notice was taken of it, and in the course of a few days he appeared to have quite recovered. Several months after severe pains were felt in the part diametrically opposite; (are we to understand the parietal bone of the other side?) and it was judged proper to trephine the bone there; but no correct information as to the true nature of the disease was obtained by the operation. After death, a fungoid mass was discovered, of the size of a large walnut, flattened, and, as it were, encysted in the diploe of the bone, where the blow had been received.

M. Velpeau has seen two cases in which blood was effused between the dura mater and bone during accouchement. It is very probable that the blood retained in this situation may undergo certain changes and ultimately give rise to some of the cranial fungoid tumours.—*Jour. Hebdomadaire.—Am. Jour. Med. Sciences.*

16. *Stricture of the Rectum treated by the Introduction of a Tent, by a New Process.* By M. Tanchou.—That there exists great analogy between stercoral and urinary fistulæ is not disputed; the following observation is a new proof of the correctness of this opinion. It also shows that the efforts of nature alone, are sometimes adequate to a cure; and that the formation of an artificial anus should be attempted, in all cases of either complete obliteration of the rectum, or of contraction of this gut to such a degree, as to prevent the escape of fæcal matter. It moreover proves, that the occurrence of a fistula, or the performance of this operation, are means by which the days of those affected with stricture of the rectum may at least be prolonged, though they may fail in producing a radical cure.

Case.—*Stricture of the rectum stercoral fistulæ caused by a gangrene of the breech; one of the fistulæ completely healed; a disposition to a complete cure brought about.*—Madame M. æt. 55, of a strong constitution naturally, and considerable embonpoint, was attacked in 1830 with a violent inflammation of the bowels. She was treated upon the antiphlogistic plan. During convalescence she indulged her appetite too freely, which brought on an attack of indigestion; in consequence of this her restoration to health was slow and imperfect, indeed it seems she never acquired her original sound constitution. In the beginning of 1831 she visited Angoulême, where she delivered herself up entirely to the dictates of her morbid and sensual appetite. In a short time she was seized with a diarrhœa, accompanied by colic and flatulency. This increased to such an extent that she had as many as forty or fifty evacuations during the twenty-four hours; to this condition of things was soon added a constant tenesmus, which obliged her to remain almost the whole time upon the close-stool. These symptoms gradually abated, without her pursuing in fact, any method of treatment; that is to say the stools were less frequent, and occasionally she was even constipated, though when this was relieved she would be attacked with violent diarrhœa and severe colic. In this condition she set out on her return to Paris, where she arrived after an extremely fatiguing and hazardous journey in the month of April, 1832, I found her very much changed in appearance, she had completely lost her embonpoint, and was very pale. Her abdomen was also considerably swollen, and she suffered from the most violent colics accompanied with a constant desire to visit the close-stool. Her attempts to evacuate were either entirely nugatory, or followed by a discharge of slimy fæcal matter, or of an extremely fœtid fluid. She still refused, as formerly, to submit to any regular treatment, particularly to a rigid course of diet. Her symptoms still increasing in violence, I determined to examine the rectum, in doing which I discovered that the anterior portion of this gut, about three or four inches above the sphincter, had undergone considerable contraction; its posterior face appeared to be as yet in a healthy condition. I insisted anew upon her observance of a rigid diet, and prescribed the application of leeches to the breech, with the design of subduing the inflammation, so that a tent might be introduced; but my orders were but partially obeyed. MM. Roux and Majendie were now called in, both of whom confirmed my diagnosis, and recommended a strict attention to the treatment which I had prescribed. Notwithstanding all this, she persevered in indulging her appetite, and could not, or would not allow the tent to remain in the rectum but a few hours at a time, and occasionally passed whole days together without its being introduced at all. The disease still continued to increase, and the cavity of the gut became smaller and smaller. By degrees she became more and more feeble; emaciation increased rapidly; her complexion became straw-colored; her abdomen remained swollen, and she evidently suffered from a retention of fæces. Enemata brought away nothing; and finally, the stricture of the rectum increased to such a degree that nothing but liquids could pass through it. She also suffered intensely from colics and tenesmus. Such was the condition of the patient in the month of January, 1833. In the month of June following, (all the above-mentioned symptoms having increased in violence,) there appeared on the right buttock a swelling, which in a short time acquired an immence size. It resembled indeed, very much, both in size and shape, a small wash-hand basin. It imparted to the touch a doughy feel, and was neither inflamed nor very sensible when pressed upon. At this period she had no fæcal evacuations at all. After a time a slight degree of redness was observed upon the surface of the tumour, together with a sensation resembling that produced by the pressure of a thin dough in the cavity of a sac, which indicated either the existense of deep-seated suppuration, or the effusion of a fluid. Positive fluctuation did not exist. The anxiety, fever, and state of desperation under which the patient at this time laboured; but more particularly the supposition that the tumour was formed by a mass of extravasated fæces, induced me to decide upon making an incision into it; accordingly one three inches in length, and corresponding in direction with the apex of the buttock, was made in its summit. This gave issue to a large quantity of very fœtid gas, fæcal matter, and a small quantity of pus; these were not encysted or collected in a mass, but seemed to be merely infiltrated. The patient was relieved, though the swelling was not sensibly diminished. On the third day after the operation, there appeared near the fold of the breech and thigh, a small gangrenous spot, which in a short time increased to such an extent as almost to touch the incisions. Thinking it the better plan to lay open this mass, I continued the first incision downwards for about three inches. The whole was then lightly dressed, after having been previously washed with chloride of lime water. The discharges from the wound were composed of fæcal matter, mixed with pus, and a gangrenous sanies. In a few days a large portion of the interior of the buttock was attacked with gangrene, and a mass of sphacelated cellular tissue, nearly equalling in size the two fists, presented itself between the lips of the wound. This was washed in the chlorine solution, and then dusted over with powdered tan-bark and quinine. The edges of the wound were dressed with strips of linen spread with storax. During this treatment, and without doubt by the efforts of nature alone, the gangrene ceased to progress; the margins of the eschar were detached; the inner surface of the buttock sloughed off, and there remained an ulcerated surface more than eight inches in diameter, and deep enough to lodge the doubled fist, which terminated above in a stercoral fistula large enough to allow the ready escape of the fæcal matters. All the symptoms produced by the retention of the fæces now ceased; the sleep became again tranquil; the appetite returned; soups and light articles of diet were easily evacuated; the fever diminished; the wound assumed a healthy appearance; granulations sprouted up from all sides; and on the 15th of August the sore had diminished at least a third; its bottom was nearly on a level with the surface of the skin; and to my great astonishment every thing indicated a speedy restoration to health.

About this time a small tumour made its appearance between the sphincter and coccyx, which soon opened; the orifice however was not sufficiently large to allow the fæces to pass readily; I therefore enlarged it. The quantity of fæces that were evacuated through the fistula, (nothing passed through the anus for two months before this time,) now began to diminish; the abdomen, which had returned nearly to its natural state, again became tense; the complexion, which had regained its clear and rosy tint, became again pallid; the colics were more frequent and violent, and she was at length obliged to keep her bed; in short, all the symptoms dependent upon a retention of fæces reappeared. I now enlarged the opening in the last tumour an inch and a half, directing the incision along the coccyx, believing that a division of the sphincter would be attended by no beneficial result. This gave issue to a large quantity of fæcal matter and pus, that had collected in this new situation. From this time forward the discharge from the original fistula daily diminished, until at length it ceased entirely, and the wound nearly healed. The injections, which formerly were discharged through several orifices, were now returned through the last incision alone. In a word, the original fistula closed, and the course of the fæces seemed to approach the natural direction. The appetite, spirits and embonpoint of the patient returned; she is also strong enough to walk in her garden, and every thing leads us to conclude, that although a perfect cure of all her complaints will not be effected, she will at least be left with merely the inconvenience of a stercoral fistula, which will not materially shorten her days. It may even be possible, after the complete cicatrization of the breech, to unite the anus to the remaining fistula, by dividing the barrier which separates the two cavities from each other, provided this barrier is not of too great an extent, and the point of communication between the fistula and cavity of the rectum is attainable with the point of the finger. The patient would then be able, after having suffered from gangrene of her breech, accompanied by several stercoral fistulæ, to evacuate her fæces per vias naturales! The manner in which the tent was introduced into the rectum during the period that Madame M. consented to its application, merits perhaps a particular notice. For the first few days it was introduced in the form of a long conical cylinder, the point of which passed through the anus, in order that this point of the gut might suffer the least degree of pressure. But in a short time the rectum, from the combined influence of the disease, and a thickening of its parietes, becoming changed both in its shape and direction, it was found impossible to follow up its sinuosities except with a stilet. It occurred to me, that by mounting the tent in the following manner, its introduction into the stricture would be materially facilitated. I had made a little tube about an inch in length, the superior extremity of which terminated in a sort of neck; its inferior extremity was conical, and opened for the reception of a forked probe about six inches in length. The tent, (méche,) supported in its middle by the neck of the tube, was so arranged as to cover the latter completely. In order to apply it, I first introduced a long flexible probe into the rectum, and searched for the stricture. Having found it, I passed up the tent by shoving it along the probe, which being held in the stricture, and its lower extremity passed through the cavity of the cylinder, served as a conductor to the point diseased. By this means the tent was safely lodged beyond the point of stricture, without the operation being attended by fatigue to the patient, and without any portion of the intestine, except the point strictured, being subjected to any distention whatever. The stilet and conductor were then withdrawn, and the tent left in the stricture. The great advantage of this sort of méche is, that it permits the escape of flatus which sometimes collects in large quantities in these cases, and is prevented from passing out by the tents usually made use of, which block up completely the cavity of the stricture.—*Gaz. Méd. de paris, Sept. 28th,* 1833.—*Am. Jour. Med. Sciences.*

17. *The Advantages of Turning the Fœtus by the Head rather than by the Feet.*—Up to the end of the 16th century, the only mode of turning ever practised was by bringing down the head first; and we find this conduct recommended, not only in such cases as are admitted at the present day to require artificial delivery, but even in common pelvic and feet presentations. Soon after the above-mentioned date, turning by the feet was first proposed, but it was not until the commencement of the 18th century that the practice was generally followed. One of the professors of the school of Strasburg resisted this innovation, strongly maintaining the superiority of the old regime; and his advice was approved of by many of the German practitioners. To justify this preference it was asserted that when the head presented first, the compression caused by the os uteri is not sufficient to injure the encephalic contents, and moreover, the communicant circulation between mother and child remains unobstructed; whereas in presentations of the lower extremities, the thoracic and abdominal viscera are exposed to a dangerous compression, and the fluids are driven back upon the head, thus causing frequently a fatal cerebral congestion. In confirmation of the truth of this statement, we are told that only one child in twenty delivered by the head is still-born; whereas, the proportion is one to five in feet presentations. In conclusion, it is alleged that whenever the fœtus is moveable within the uterus, it is quite as easy to effect the turning by the head as by the feet.

M. Dubois dissented from the above arguments. He contended that the described dangers of any compression on the abdomen and thorax were most unnecessarily exaggerated, and instanced two cases wherein the shoulder presented along with the head, and yet the children were delivered without any contusion of the thoracic and of the abdominal viscera.

The dread too of the retropulsion of the blood upon the head was an offspring of fancy rather than a result of experience, he did not agree with them in their belief that the os uteri exercised such a consecutive pressure as was alleged; the parts of the fœtus which have already escaped from the uterus are subjected to a less degree of pressure than those still contained within its cavity; and hence we can readily explain why the blood should be driven to and accumulated in the former. Do we not observe that when an arm is born first, the member frequently becomes much swollen? now this swelling arises from the pressure being less upon the arm than upon the rest of the body. True it may be, that in many children who die after feet presentation, visceral congestions are not unfrequently discovered; but the cause of these is the compression of the umbilical cord, and not the retropulsion of the fluids which M. Flamant believed to take place.

The compression of the cord is a necessary danger attending all births by the feet, and indeed it constitutes a very serious objection to the process of turning; the child is very often asphyxiated, and in such a case we find upon dissection the same phenomena which are observed after drowning or hanging, viz., an apoplectic plethora within the head, great congestion in the veins of the cerebrum and other viscera.

The calculations which have been adduced to prove the greater safety of turning by the head than by the feet, are not strictly correct, as will appear from the following statement of M. Dubois.

In all such calculations, to ascertain the comparative mortality of the different modes of delivery, we must be careful to exclude from our tables all cases wherein the child has died before actual accouchement has commenced; or wherein the labour has been premature, and the child may be therefore not well capable of independent life. Now the new tables which have been recently formed at the Maternité of Paris, on these principles, show, that from the 1st of June, 1829, to the 1st of June, 1833, 10724 children have been born at the hospital; of these 10262 were born by the head, 391 by the lower extremity, 59 by the trunk, and—30 by the face; of the 10262, 9867 were at the full period of gestation, and 395 were not. The 9867 may be reduced to 9837, because, in 30 of the cases the fœtus was known to be dead before delivery commenced, and the 395 premature cases may be reduced to 278; for in 83 the fœtus had been dead for some time, and in 34 it was too imperfectly developed for the maintainance of independent life.

Of the 9837 deliveries by the head at the full time, 191 were born dead; the proportion is therefore one in 51 or 52; and of the 278 prematurely born, 48 were born dead, or one in every 5 or 6. Of the 391 deliveries by the lower extremity 238 were at the full term, and 153 before the term; from the first number we must deduct 7, who were dead before labour began; and out of the remaining 231, 21 were born dead; a proportion of one to eleven. From the 153 we must deduct 63, in which the child had evidently died during pregnancy, and 30, in which it was too young for independent life; and out of the remaining 60, 10 were born dead; or one in six. From these calculations it appears among other results, that fœtus at the full period can endure the "*fatigues of accouchement*" with much greater safety than when born at an earlier period, whether they are delivered by the head or not. M. Dubois draws our attention to the important difference in the results by the previous deduction of all the cases in which the fœtus either had been dead for some time before labour, or was incabable of life when delivered. Thus had we enumerated these cases among the mortality in the 10262 head presentations, we should have had 386 deaths, or one in 25; whereas we have fixed it above at one in 51: and in the 391 feet presentations the deaths would have amounted to 134, or nearly one in two instead of one in eleven. With regard to the comparative advantages in practice of turning by the head, M. D. admits that in some cases the operation is not quite possible, [Mad. Lachapelle was wrong in denying this,] but also abundantly easy. He has himself performed it twice when the shoulder presented; but the operation is much more difficult than that of turning by the feet, and should the liquor amnii have copiously escaped, or should the uterus be firmly contracted around the child, the manœuevre is almost impracticable. In the 59 trunk presentations, two were delivered by means of turning by the head; in a third case the expulsion of a putrid fœtus took place by the shoulders; and in the remaining 56 the child was brought down by the feet. Out of the whole number 59, in 25 only did the child survive; but M. Dubois is of opinion that a still smaller number would have been saved had turning by the head been tried in all.—*Med Chirurg. Rev., January,* 1834.—*Am. Jour. Med. Sciences.*

18. *Clinical Observations on Fractures of the Neck of the Femur.*—By Baron Dupuytren.—*Age at which such fractures occur.*—Fractures of the neck of the femur have attracted so much attention of late years, that we are told in most *ex professo* treatises on such subjects, that they are as well understood as those of other parts of the thigh-bone. Those, however, who have witnessed the practice of the Hotel Dieu during the past winter, must be aware that much additional light has lately been thrown upon the subject. If you examine, said M. Dupuytren, the age of different individuals who are at this moment in our wards with fractures of this part, you will perceive that they are all past 50 years of age; and among those to be hereafter pointed out to you, you will remark that there are no children, and in general very few adults. But in both sexes you will find this accident more common after 60 years of age. Never in any instance were the predisposing and exciting causes more conspicuous than here. I have never seen fracture of the neck of the thigh in an infant, and very rarely in a young person. Sabatier, however, in his interesting memoir in the transactions of the old Academy of Medicine, mentions the case of a youth of 15, who had a fracture of this kind. These lesions, on the contrary, become more frequent as life advances, and are most common between 70 and 80 years of age. It is impossible but that there must be some cause for this difference in the period of fracture of the neck of the femur at different epochs of life.

*Predisposing cause.*—This cause is well ascertained, and resides in the anatomical disposition of the parts, which is not the same at different ages. The neck of the femur, in fact, has not at all periods of life the same direction; and this disposition of the parts is important to be known. In early life the axis of the neck approaches to that of the body of the femur; the angle which it forms being the most open possible. The great trochanter makes but a very small projection; and we shall see by and by that falls upon this part are the most frequent cause of fracture of the neck of the femur;—that the frequency of the accident is in direct proportion to the extent to which the trochanter projects;—and that such projection is in direct proportion to the length of the neck of the bone, and the greater or smaller angle which it forms with the bone. Now we know that the trochanter lies deep in children, and that it hides itself, so to speak, behind the prominence of the os coxæ; the result of this is, that in falls on the side, the blow does not come upon it, and consequently the risk of the fracture is diminished. Another anatomical disposition renders fracture of the neck still more difficult. The shorter the bone is, the less of a right angle it makes with the body, and consequently the more it approaches the direction of the axis of the femur, and the causes of the fracture have therefore less influence on the neck. There is also another reason why fractures of the neck of the femur are rare at an early age—namely, the great flexibility of the bony tissue, in consequence of the abundance of organic matter in the bone. In adult age, fracture of the neck of the femur is rare, but less so than in infancy; the earthy matters are more abundant than at an earlier age, but less so than they subsequently become. The neck also presents a different disposition: it is larger, and the angle which it forms with the body is much more marked than in the child. There results from this more projection of the great trochanter, and consequently more purchase for the causes tending to produce fracture, to act upon. This greater length of the neck of the femur, however, and this projection of the trochanter, vary further according to sex and individual peculiarities. Women have the neck of the femur longer, and consequently a more projecting trochanter, than men. The nearer, in the male sex, the formation approaches to that proper to the female, the more risk is there of this fracture.

If the relief of the muscles of the haunch in men presents an obstacle to the production of fracture, by diminishing the effect of falls upon the great trochanter, the thickness of the layer of fat in women fulfils the same indication: but when there is much emaciation present, women become more liable than men to the accident. Let us next inquire how it happens that old men are so much exposed to this kind of fracture. At this period of life the pelvis has acquired all its breadth; the trochanter major is more projecting; the neck of the femur longer, and inclined to a right angle. Besides, the skeleton of the old man weighs much less than that of a young adult, which depends upon the bones having lost much of their organic substance,—having a greater quantity of earthy matters, and being thus also more friable. In aged women, while the anatomical disposition retains the peculiarities formerly mentioned, the friability is even more marked than in men. Accordingly, at the Salpetriére, which is an asylum for old women, there are more cases of this nature than at the Bicetre, which is a receptacle for old men. These considerations are of importance in reference to the theory of fractures. Thus in the child the cure may be effected in three weeks or a month, while in the adult it will require a much longer period; and in those advanced in life, a hundred or a hundred and twenty days are required.

If we recapitulate briefly what we have said with respect to the predisposing causes of this fracture, we shall see that shortness of the neck of the femur, very considerable openness of the angle which it makes with the body, deficiency in the projection of the great trochanter, flexibility of the osseous tissue, and abundance of adipose matter, render it almost impossible that fracture of the neck of the femur should take place in children. In women, the less considerable obliquity of the neck relatively to the axis of the body of the bone, the length and prominence of the great trochanter, explain the greater frequency of this accident in them than in men. Sir Astly Cooper has said that fracture of the neck of the femur rarely took place before the age of fifty; but there are many exceptions to this rule. There are also some other particular circumstances which predispose to fracture of the neck of the femur—such as rachitis and cancerous diathesis; but as these are common to all parts of the osseous system, it is unnecessary to dwell upon them here.

*Exciting causes.*—What, then, are the exciting causes of this fracture? Almost all the patients whom we interrogate reply that they have fallen on the great trochanter, and in such manner that they have been unable to protect themselves with the arm, as is usually done instinctively. The frequency of this cause has been recognized by all authors. Thus of thirty-six cases observed by Dessault, twenty-four were produced by falls of this kind. In infants and young persons who have fallen on this part, and who have been preserved from fracture, we meet with decollation of the epiphysis. Falls upon the trochanter are, however, far from being the sole cause of fracture of the neck of the femur; I shall point out others which play an important part: but I must first make a remark which has excited little attention; namely, that at the same time the patients fracture the bone, they bruise the external parts. I this morning examined a woman who stuttered, and answered badly the questions put to her; but on touching the great trochanter it gave her much pain, although the limb was not moved; and this led me to examine the part, when I found a large ecchymosis upon it. This fact is of some importance in directing us to the part which the patient has struck, when he is himself unable to give an account of his fall.

I have said that other causes might produce fracture of the neck. This is, in fact, what happens in a fall on the soles of the feet, or still more upon the knees; but in both cases it is necessary that the muscles be stretched and flexible. Sir Astly Cooper has observed that in London, fracture of the neck of the femur often arises from a false step off the edge of the pavement. Under such circumstances the head of the femur impinges strongly against the cotyloid cavity: from this there results an effort tending to diminish the angle which the neck makes with the body of the femur. I have also frequently observed a depression of the cotyloid cavity produced by the head of the femur, in consequence of a fall on the feet or knees.

It is not from this cause, however, but from a fall on the haunch, that this accident generally happens. In this case the neck is placed between two opposite forces; the head of the bone being pressed on the one side by the weight of the body, while the great trochanter is opposed by that against which it strikes, and the point which most usually gives way is that immediately under the head of the bone, at the upper and internal part of its neck.

It has also been thought that this fracture may arise simply from a muscular exertion, and a case is quoted in which this is said to have taken place in a negro affected with tetanus.

This fracture may likewise arise from causes directly applied. These are usually projectiles—such as cannon-balls, several instances of which I saw after the days of July.

*Diagnosis.*—The diagnosis of this kind of fracture is not made without difficulty. It sometimes happens that a fall on the haunch, accompanied by bruising of the muscles and joints, imitates this fracture; while, on the other hand, the same cause may produce an actual solution of continuity, and yet the patient be able to run and walk. It is thus that persons have been known, after they had fractured the neck of the femur, to be able to reach home without presenting any shortening: it is thus that displacement of the fragments does not take place till after some hours, or even many days, either in consequence of some movement on the part of the patient, or of the examination made with a view of ascertaining the nature of the malady. Before we proceed, let us say a few words with regard to the cause of this consecutive displacement.

*Secondary displacement.*—It is known, that at the first period of its formation, the callus of long bones often yields, and produces deformities, when a perfect cure has been expected. Who has not seen oblique fractures of the femur give way under weight impossed upon it when the patient began to walk again, at a time when all risk seemed to be at an end? The same thing happens in fractures of the neck of the femur. At the end of two or three months the provisional callus yields beneath the weight when the patient rests upon it, and shortening is the result. I have seen this at the end of two, three, and four months. It is therefore necessary that patients should be controlled by the proper apparatus, during 100, 120, or 150 days, or even more.

The weight of the limb, and still more that of the body, on the broken part, is to be regarded as the great cause of displacement, whether primitive or consecutive. But another active power in producing displacement consists in the prolonged action of the cause which has given rise to the fracture. It sometimes happens that it buries the upper fragment in the thickness of the spongy tissue of the superior extremity of the lower fragment, and consolidation takes place in this situation rather quickly. Numerous anatomical preparations in the museum of the Hotel Dieu exhibit this phenomenon, and sufficiently demonstrate the reality of its existence. Lastly, there exists another cause of displacement in fractures in the neck of the femur,—namely, muscular action.

The primitive symptoms take place when, in a fall upon the heel or the knee, the shortening and displacement take place at the moment. In this case it is clear that, the upper fragment remaining in its place, the lower one is pushed up by the weight of the body. But a vertical fall is the least common cause of fracture of the neck of the femur; and when, as is most common, the blow is received on the trochanter, the cause tends not to shorten but to lengthen the limb. There exists, then another cause of this shortening, which has until now been but little understood. It depends upon the adductor muscles, which, being designed to carry the limb outwards when it has rotated, are inserted on one side in the ischium, and on the other terminate behind and along the linea aspera of the femur. It is on these that the displacement, and, in part at least, the shortening depend.

When there is displacement, the fracture is always easily recognized; but when this is not the case, the nature of the mischief may be suspected—without, however, being placed beyond doubt. I suppose that the symptoms are well characterized; that there is shortening, dispacement of the limb outwards, and inability on the part of the patient to raise himself: it is necessary then to inquire if the limb preserves this shortening, or may be made to lose it by extension, and if the great trochanter turns on the axis of the femur, or on the extremity of the lever.

If the shortening is only some lines, it is difficult to distinguish it from that which is produced by an upward movement of the pelvis, caused by the contusion: the diagnosis becomes more evident if it extends to half an inch or more. Displacement, however, may also depend on luxation of the head of the femur, or upon an ascent of the pelvis. In luxation forwards, the head of the femur passes on the horizontal branch of the os pubis—then there is shortening; but the cause is detected by the hard tumor which may be felt to roll when the femur is moved. In luxation into the sub-pubic region the member is also turned outwards, but it is elongated; and there is in this situation an enlargement and unusual tension of the muscles.

In luxation upwards and outwards, the head of the femur is in the external iliac fossa; the limb is shortened, but the point of the foot and the patella are turned inwards, the heel being thrown out.

There is, finally, a luxation downwards and backwards, which I have only observed two or three times: the limb is then turned inwards, and sometimes a little elongated, and it cannot be brought into its ordinary state except by the effort of reduction, and, once reduced, the displacement does not again recur. Thus, the distinctive character is as follows:—shortening produced by a fracture yields to the smallest effort at elongation: shortening produced by a dislocation is more difficult to remove, but, once reduced, the deformity disappears.

Let us now enquire what are the material effects of these fractures upon the bones, beginning with the glenoid cavity. I have found this cavity driven in several times by the head of the femur: this accident was produced by a fall on the feet or knees. In this case, the head of the femur impinges with violence against the bottom of the cavity, and, being the more resistant, breaks it. The most remarkable case which I have observed was this: the bottom of the glenoid cavity had been driven in, and the head of the femur, which remained entire, had passed completely into the pelvis; the neck, which had not experienced any solution of continuity, was so firmly locked in this accidental opening, that it was very difficult in the preparation to disengage it, and thus reduce this new kind of dislocation. In other cases, the cotyloid cavity is broken without the head of the bone being displaced; but the most common effect of the fracture is to be seen at the upper extremity of the femur in the radiated comminution of the head of the bone, the neck remaining entire. The most common cause producing this accident, is a gun-shot wound acting directly on the part; though it also arises from falls on the great trochanter, and even on the soles of the feet.

The neck, however, is much more frequently the seat of fracture, because it forms a lever. Its diminished size towards its middle part also contributes to the production of the fracture. This may take place from below upwards, or from above downwards, depending upon the manner in which the fall occurs; but usually it is at the base of the neck that the fracture takes place: there are, however, infinite varieties in this respect.

*M. Dupuytren's Opinion of Sir Astley Cooper's Views.*—I request you to observe, that according to the seat of these fractures they are called intra-capsular, or extra-capsular; and it is a distinction which has been much dwelt upon, because many practitioners think that it is difficult, or impossible, that the consolidation of the fracture can be effected when it has taken place within the capsula; while they admit that it is possible, and even easy, when it has taken place without the capsula. Astley Cooper, whose authority is so imposing in surgery, says expressly, that in all cases of transverse fracture of the neck of the femur within the capsular ligament, which he has had occasion to examine, he has never found bony callus; and he is persuaded that it is impossible; he has also made experiments upon living animals, which have confirmed him in his opinion; and the English surgeons have equally adopted the doctrine of their countryman.

But to the facts which they adduce in support of this non-consolidation, numerous facts of an opposite nature may be advanced. A considerable number of anatomical preparations show intra-capsular fractures exceedingly well united; and those which exist in the cabinets of the faculty at Paris, and the school at the Hotel Dieu, prove that this consolidation, with or without deformity, is real. Sir Astley Cooper has probably only seen fractures of the neck of the femur which have not been cured, which have been treated ill, or not treated at all. This is the only way of explaining the opinion of the English surgeon, which is evidently erroneous. An examination, however, of these anatomical preparations, though evidently calculated to convince us of the reality of this union of fracture within the capsule, does not appear to have produced this effect on the other English surgeons who have visited our museum. Mr. Cross says he has attentively examined the preparations in the School of Medicine at Paris, and that none of them appear to him calculated to prove that bony union ever takes place, when the head has been completely separated from the capsular ligament. When one has seen the preparations at the Hotel Dieu, which every person may examine at leisure, if he then denies the possibility of consolidation within the capsule, of a surety I know not of what nature the proofs would require to be to produce conviction. For myself, I regard this consolidation as demonstrated, despite of what the English surgeons say.

*Of the indications of Cure in Fractures of the Neck of the Femur.*—At first sight one might suppose that it was the same in fractures of the neck of the thigh-bone as in other solutions of continuity—that it was sufficient to replace the fragments and maintain them in contact. But how many difficulties present themselves? How are fractures of this kind to be reduced? Are we to use powerful extension and counter-extension? Certainly not; because we should thus increase the tension of the muscles, which are already very great. In luxations this may be effected by diverting the attention of the patient, and seizing that moment to produce the reduction. But in fractures this cannot be done, because the attention of the patient is concentrated on the injury. Another method of overcoming muscular action is to place the limb in a state of flexion, as recommended by Pott in fractures generally. I believe I am the first who applied these rules to fractures of the neck of the femur. I suppose, that, in a case of this kind, extension and counter-extension have been used: it is evident that, if the limb be placed in a state of flexion, no difficulty is experienced in overcoming the displacement as to length and turning. But how are the fragments to be kept in contact? I suppose that the fracture of the neck of the femur is transverse, and that this part is divided into two halves; it may then easily be reduced; but the difficulty is to maintain it so, because the fragments are not placed fronting each other. Suppose, now, that the fracture is oblique; if the patient attempts to walk, the lower fragment impinges slightly upon the upper one: this fragment, then, will offer some obstacle to its displacement; so that this is less easy than in a perpendicular fracture.

The general indication, then, is to reduce the fragments, and keep them in contact; and the principle established by Pott fulfils this purpose, by preventing the muscles from contracting; but, in order to obtain consolidation, it is necessary to employ at least twice as long a time as in fractures of the body of the bone; while it is necessary to add twenty, thirty, or forty days more, in order to obviate the risk of displacement occuring when the patient begins to walk. If this treatment be pursued, I venture to assert that secondary displacement scarcely ever occurs.—that, when it does happen, it is extremely slight.

The best method of effecting reductions of fractures in general, and that of the neck of the femur in particular, is to diminish the resistance of the muscles, by placing them in a state of relaxation or demi-flexion, as you see us do every day, in the following manner:—The patient being laid on his back, and his pelvis fixed by means of assistants, the thigh is bent on the abdomen, by raising it, and making moderate traction; the limb is also bent upon the thigh. Scarcely is this done, when, without difficulty and without effort, the limb resumes its ordinary length, and the foot its natural direction.—*From the "Leçons Orales," published periodically, under the Baron's inspection.–Lond. Med. Gaz.–Bost. Med. and Surg. Jour.*

19. *On the Prevention of Uterine Hæmorrhage, Post Partum.*—By Dr. Beatty.—In our esteemed contemporary of Doublin, for January of the present year, there are some "contributions to midwifery"—(a somewhat equivocal term, by the bye, from the fertile soil of the Emerald Isle)—by Dr. Beatty, consulting accoucheur to the Baggot Street Hospital, Doublin. The one which we shall now notice is on a very important subject. Uterine hæmorrhage, post partum, is a formidable accident. The gush of blood, on such occasions, strikes terror into the minds of all. The loss of the vital fluid, in the greatest operations, is comparatively trifling to this. It would seem that the parturient woman bears better an excessive hæmorrhage than any other individual—probably in consequence of the habit so long sustained in the system, of devoting a large portion of blood to the purpose of utero-gestation. Still there is a limit to this tolerance of hæmorrhage, beyond which a female cannot go in safety, though some bear a much greater loss than others. This hæmorrhage can result, according to our author, from one cause only—viz., a patulous state of some or all of the great uterine sinuses, in consequence of want of contraction. "The only remedy for this is a proper contraction of the fibres through which these vessels pass obliquely." Uterine contraction, therefore, is the only protection against uterine hæmorrhage; and will always be effectual except in cases of morbid adhesion of the placenta. The best means of effecting this contraction, according to Dr. Beatty, is direct stimulus by external force, viz. grasping, friction, and firm pressure over the pubis. Dr. B. says that this was the practice pursued by his father, with great success, during a long practice of 45 years. He does not wish, however, to discard other assistance, as cold, &c. He only desires to place the above means in the first rank.

"Early impressions are very lasting, and therefore I have a vivid recollection of the first case of serious uterine hæmorrhage I ever witnessed. I was called in the middle of the night to a patient, who had been attended by a very young man, a student of midwifery. The labor had been natural and easy; but after the birth of the child, and before the expulsion of the placenta, a deluge of blood escaped; and when I arrived, there was not only a sea of it under the patient, but also a stream along the floor, that had issued from the foot of the bed. I found the attendant pale as a corpse, and almost frightened to death, with a bucketful of water beside him, and numerous cloths soaked in the same, which he diligently applied to the external parts; notwithstanding which, the bleeding still continued. The woman was blanched, the pulse failing at the wrist, she was tossing her arms about, and crying out for more air. On passing my hand over the abdomen, and feeling the uterus large and flaccid, I immediately exerted all my force, in grasping, and firmly pressing this organ downwards into the pelvis, and very soon found it contracting forcibly under my finger. At this moment a rush of coagulated blood took place, which nearly extinguished the little remaining spark of life in the attendant, but was a matter of great consolation to myself, as I took it as a token of having succeeded in my endeavors. In this I was not deceived; the uterus had fairly contracted, and the hæmorrhage was at once arrested. I kept up the pressure on the uterus with my left hand, and passed the forefinger of my right into the vagina, to ascertain the state of the placenta, which I found now lying loose in that passage, from whence, after having put on a tight binder, it was easily removed. The woman recovered; but she had lost so much blood, that some days elapsed before she could be pronounced out of danger."

Dr. B. conceives that, in the foregoing case, the introduction of the hand into the uterus would have been productive of greater loss of blood than the procedure which he employed. If, however, he had found the natural contraction of the uterus insufficient to expel the placenta, he would then have had recourse to extraction.

As we cannot prognosticate whether or not hæmorrhage will take place, post partum, it is better to guard against its occurrence. "This is attained by making the uterus perform the whole process of expelling the child itself, even to the feet; and never, by any injudicious haste, assisting the delivery by pulling the child."

"A practice pretty generally employed in this city, and lately taken notice of by Dr. Maunsell, is of great utility in this part of the process; that is, after the expulsion of the shoulders, to place the left hand on the abdomen of the woman, and follow the uterus by firm pressure, until the whole child is expelled. After this has taken place, if the child be alive and cry, the right hand, which had been employed in supporting its head and body, may now be disengaged, and the child laid in the bed, until more important matters are attended to. The chief of these is the proper application of an appropriate binder, previously passed loosely round the body of the woman. This I consider a very important part of the treatment, for it at once insures an equal and firm pressure on the uterus, and prevents its subsequent relaxation; while it leaves the practitioner at liberty to attend to the child. But the kind of binder usually employed, is very ill calculated to accomplish this end. It is commonly made of some straight narrow material, as a folded towel, a piece of linen, or what is still worse, of flannel, any of which it is utterly impossible to apply in such a manner as that it shall keep its place, and exert the uniform pressure which is so desirable; as from the shape of the woman's body, it must slip over her hips, and it finally runs into a simple cord round her waist, no matter how broad it may have been, or how accurately it may have been at first fastened.

"To obviate this difficulty, I make all my patients provide themselves with a binder, according to a pattern which I have constructed, and have found of the greatest use and convenience. It is made of jean, or twilled calico, doubled, and broad enough to reach from the eighth or ninth rib to the trochanters; with two long triangular pieces, termed in milinary, gores, let in to enlarge the diameter below, and fit the hips, just as female stays are made. It is furnished with a row of buckles arranged along one end, and at the other with a corresponding number of straps, made of the same material as the binder. The straps are about seven inches long, and are sewed, not to the edge, but about seven inches from it; so that when they are passed through the buckles, the floating portion passes under the opposite end, and protects the skin from pressure. A very thin piece of whalebone, one-third of an inch broad, is inserted, so that when the binder is applied, it runs straight down the middle of the abdomen from the thorax to the pelvis. A bandage such as this fits easily, without any unequal pressure when drawn tight; never shifts its place when made well, and properly applied; and effectually accomplishes the object for which it is intended. I have employed it with several ladies who had been in the habit of using the common kind, and they invariably express the greatest comfort from its use."

As soon as the child is expelled, and when the uterus is felt to be well contracted, the binder may be tightened. It is best to begin with the middle straps, and proceed regularly downwards, after which the upper may be secured. The course thus pursued is admirably calculated to prevent the occurrence of the hour-glass contraction—and the best security against the insidious, and too frequently fatal accident, relaxation of the uterus after delivery, accompanied by internal hæmorrhage.—*Med. Chir. Review.—Bost. Med. and Surg. Journal.*

20. *On the Effects of Mammary Irritation in Amenorrhœa.*—By Charles Patterson, M. D.—Mary Reardon, æt. 24 years, of moderately corpulent habit, was admitted into the Rathkeale Hospital on the 10th of August, 1832. She laboured under slight synochial fever, which, in a few days, yielded to venesection and purgatives. On the 19th August, symptoms, which were considered of a hysterical character, presented themselves, with pain in the upper and outer part of the right side of the chest. For the latter affection a small sinapism was prescribed, but from inattention of the nurse, it was made so large that it covered a considerable portion of the mamma. The sinapism remained on for half an hour.

At the visit on the following morning, the 20th August, Reardon complained that the right breast was exceedingly painful, the pain being very different in its character from that which she had before experienced. On examination, the whole side of the chest was found considerably swollen; there was slight diffused redness of the skin; and though the mamma itself was enlarged to four or five times its natural bulk, yet there was no circumscribed hardness, nor any tendency to suppurative inflammation.

On the 21st August, the right mamma and adjoining parts of the chest, were found much more enlarged than they had been at the preceding visit. The left mamma and side of the thorax were unaffected, and it was announced by the nurse, that the catamenia had that morning appeared, and were then present in considerable quantity.

The discharge, which, as the patient stated, had been for two years and a half wholly suppressed, continued to flow for two days; then it began to decline, and with it the tumefaction of the mamma gradually disappeared.

My attention having been thus accidentally directed to the practicability of exciting the torpid functions of the uterus, through mammary irritation, I availed myself of the next opportunity presented to me of again observing the effect of that operation.

Catherine Power, æt. 19 years, applied to me the 14th September, 1832, she complained of headache, languor, loss of appetite, and inability to attend to her usual business, that of a servant. She stated that about the middle of April, the menstrual discharge being then present, she incautiously exposed herself to cold in washing clothes at a river. The catamenia then suddenly ceased, had not since returned, and from that period she had been constantly subject to ill health. She had consulted different medical gentlemen, and taken a great variety of medicine with little advantage.

I directed that the clavicular half of the right mamma should be covered with a sinapism. It was allowed to remain on for thirty minutes; and on visiting her in six or seven hours after its removal, I found the whole right breast considerably swollen, hot, and painful. The next morning the enlargement of the mamma was very much increased, the tumefaction having extended to the clavicle and axilla of the irritated side. There was no hard circumscribed or prominent tumor, but a painful diffuse elastic distention of the mammary gland and surrounding cellular substance. On the evening of the day next succeeding the application of the sinapism, this poor girl, with much joy, reported that the catamenia had appeared. The flux having continued for two or three days in moderate quantity, she then found herself greatly relieved of the headache and other most distressing symptoms; and in a week her health was so far restored, that she ceased to require any further attendance.

In both these cases cold evaporating lotions and gentle saline aperients were employed to moderate the local phlogistic engorgement. Both patients have since continued to menstruate at the regular periods.

From the facility with which the menstrual flux was induced, in the preceding cases, it would seem that the beneficial effects, in amenorrhœa, lately observed to arise from the long continued daily application of one or two leeches to the breasts, were entirely owing to the great irritation which the leech bites had eventually produced in these organs. The abstraction of blood by leeches from the mamma, had not, according to the reports of the cases in which they were employed, the least perceptible influence over the uterine functions, until pain, heat, and excessive tumefaction of the breasts had been first developed. Phlogistic engorgement of the mammæ being then the essential step in the movement, which, in these instances, determined the flow of the catamenial discharge, it must be obvious, that for the production of the necessary irritation to effect that engorgement, the simple application of a sinapism would have been, in every respect, infinitely preferable to the tedious and troublesome process of the daily repetition of leeching.

But it must not be supposed that mammary irritation is applicable to every form of amenorrhœa. I know that it will not be successful in every case, for I have found it to fail.

Mary Fitzgibbon, æt. about 21 years, of spare habit, was affected with headache, and irregular dyspeptic symptoms. The headache permanent, with occasional aggravation; countenance and tongue chlorotic; mammæ undeveloped. The menses had been scanty and irregular from the 16th to the 19th year of her age, but during the last two years they had been totally suppressed. No apparent organic impediment.

A sinapism was first applied to one breast, and afterwards a similar application was made to both breasts at the same sime. But though the sinapisms produced their ordinary effects, considerable pain and cutaneous irritation, yet the enlargement of the mammæ was very trifling, and there was no consequent uterine action.—*Dublin Jour.*, *Nov.* 1833.—*Balt. Med. Sur. Jour.*

